# First monotreme from the Late Cretaceous of South America

**DOI:** 10.1038/s42003-023-04498-7

**Published:** 2023-02-16

**Authors:** Nicolás R. Chimento, Federico L. Agnolín, Makoto Manabe, Takanobu Tsuihiji, Thomas H. Rich, Patricia Vickers-Rich, Fernando E. Novas

**Affiliations:** 1 Laboratorio de Anatomía Comparada y Evolución de los Vertebrados, Museo Argentino de Ciencias Naturales “Bernardino Rivadavia” (CONICET); Av. Ángel Gallardo 470, C1405DJR Ciudad Autónoma de Buenos Aires, Argentina; 2 Fundación de Historia Natural “Félix de Azara”, Departamento de Ciencias Naturales y Antropología, CEBBAD - Universidad Maimónides, Hidalgo 767, C1405BDB Buenos Aires, Argentina; 3 National Museum of Nature and Science, 4‑1‑1 Amakubo, Tsukuba, 305‑0005 Japan; 4 Department of Earth and Planetary Science, The University of Tokyo, 7‑3‑1 Hongo, Bunkyo-ku Tokyo, 305‑0005 Japan; 5 Museums Victoria; P. O. Box 666, Melbourne, Victoria 3001 Australia; 6 School of Earth, Atmosphere and Environment, Monash University, Clayton, Victoria 3800 and Department of Chemistry and Biotechnology, Swinburne University, Hawthorn, Victoria 3122 Australia

**Keywords:** Palaeontology, Phylogenetics, Zoology

## Abstract

Monotremata is a clade of egg-lying mammals, represented by the living platypus and echidnas, which is endemic to Australia, and adjacent islands. Occurrence of basal monotremes in the Early Cretaceous of Australia has led to the consensus that this clade originated on that continent, arriving later to South America. Here we report on the discovery of a Late Cretaceous monotreme from southern Argentina, demonstrating that monotremes were present in circumpolar regions by the end of the Mesozoic, and that their distinctive anatomical features were probably present in these ancient forms as well.

## Abstract (web summary)

An isolated tooth from the Late Cretaceous of Argentina indicates a monotreme presence in South America by the end of the Mesozoic.

## Introduction

The fossil record and extant distribution of monotremes is almost restricted to Australasia, with the single exception of a fossil ornithorhynchid from the earliest Cenozoic in Patagonia^1^. In this context, occurrence of a monotreme in Patagonia was interpreted as the result of a single dispersal from Australia to South America, before or during the Late Cretaceous or early Paleocene^2–6^.

The aim of present contribution is to report on the discovery of a new monotreme, *Patagorhynchus pascuali* n.gen. et sp., which represents the first Cretaceous toothed monotreme from Gondwana. The material here reported consists of a fragmentary right jaw preserving the lower molar 2 showing the dilambdodon pattern characteristic of monotremes. The molar was collected from levels of the Chorrillo Formation (Upper Cretaceous, Early Maastrichtian^7^), cropping out in SW Santa Cruz province, Patagonia, Argentina. It was found in association with both terrestrial and aquatic mollusks, calyptocephalellid anurans, chelid turtles, snakes, ornithopod, sauropod, and non-avian and avian theropod remains^7,8^. As far as mammals is concerned, the same fossil spot yielded a molar of the gondwanatherian *Magallanodon baikashkenke*^9^ and isolated caudal vertebrae regarded as Mammalia incertae sedis^8^. From almost equivalents levels belonging to the Dorotea Formation (Valle del Río de las Chinas, southern Chile), remains of *Magallanodon* and the meridiolestidan *Orretherium* have been reported^10,11^.

## Results

Mammalia, Linnaeus 1758

Monotremata, Bonaparte, 1837

*Patagorhynchus* gen. nov. (monotypic genus)

### Etymology

*Patago*, from Patagonia, and *rhynchus*, nose.

### Diagnosis

*Patagorhynchus* differs from basal monotremaformes (including *Steropodon*) in having a dilambdodont crown morphology and a labial cingulid;^12^ dilambdodont disposition of cusps and crests on molar crown is shared with *Teinolophos* and ornithorhynchids; *Patagorhynchus* and ornithorhynchids differ from the basal monotremaformes *Teinolophos* in having notably low and mesiodistally expanded teeth with the anterior lobe (equivalent to trigonid) positioned lower than the posterior one (equivalent to talonid), in having talonid composed of two (rather than one) transverse lophids, and lacking a labial cingulid. The anterior cingulid of *Patagorhynchus* is wider than that in *Teinolophos* but narrower than that in *Obdurodon*. *Patagorhynchus* shares with the toothed monotremes *Obdurodon* and *Monotrematum* both lingual and buccal extremes of the V-shaped lobe (equivalent to trigonid) with one buccal and two lingual cusps, with the first being more elevated than the latter two, and a complete mid-valley. *Patagorhynchus* bears two roots on m2 (as also probably in *Monotrematum*) and differs from *Obdurodon* and *Ornithorhynchus*, in which more than 5 roots are present. The lobes of *Patagorhynchus* and *Obdurodon* show hypsodonty, in contrast with the much more brachyodont molariforms of *Monotrematum*. *Patagorhynchus* exhibits the following features that are lacking in other monotremes, and may be considered autapomorphic among monotremes: mid-valley labially diverges (i.e., the length of the labial edge of this valley represents two times its lingual length) and anterior cingulid labially narrow and does not reach the labial margin of the protoconid.

### Type and the only species

**Fig. 1:**
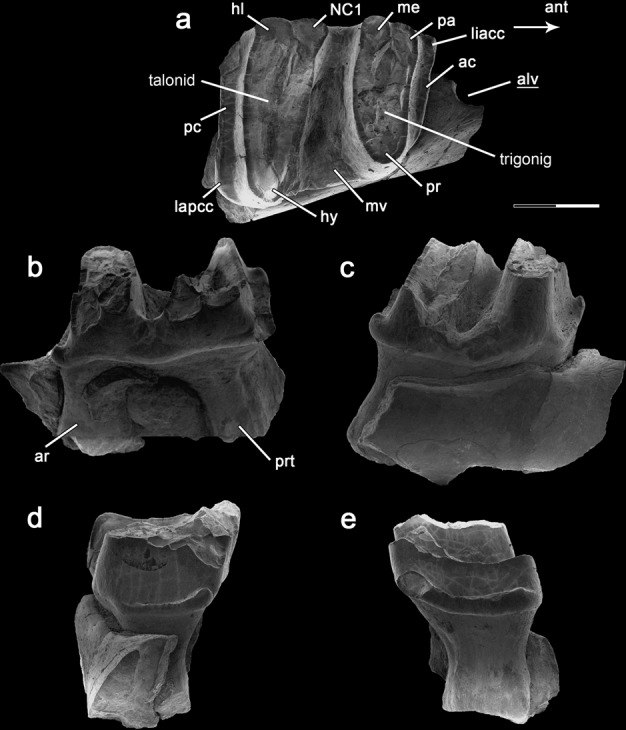
Images of *Patagorhynchus pascuali*, MPM-PV-23087. Lower molar 2 and part of the right jaw, in **a**, occlusal view; **b**, medial/lingual view; **c**, lateral/labial view; **d**, posterior view; **e**, anterior view. Scale bar: length 2 mm. Abbreviations, ac, anterior cingulid; alv, alveolus; ant, anterior; ar, anterior root; hy, hypoconid; hl, hypoconulid; lapcc, labial posterior cingular cusp; liacc, lingual anterior cingular cusp; me, metaconid; mv, mid-valley; NC1, neomorphic cusp 1; pa, paraconid; pc, posterior cingulid; pr, protoconid; prt, posterior root.

**Fig. 2:**
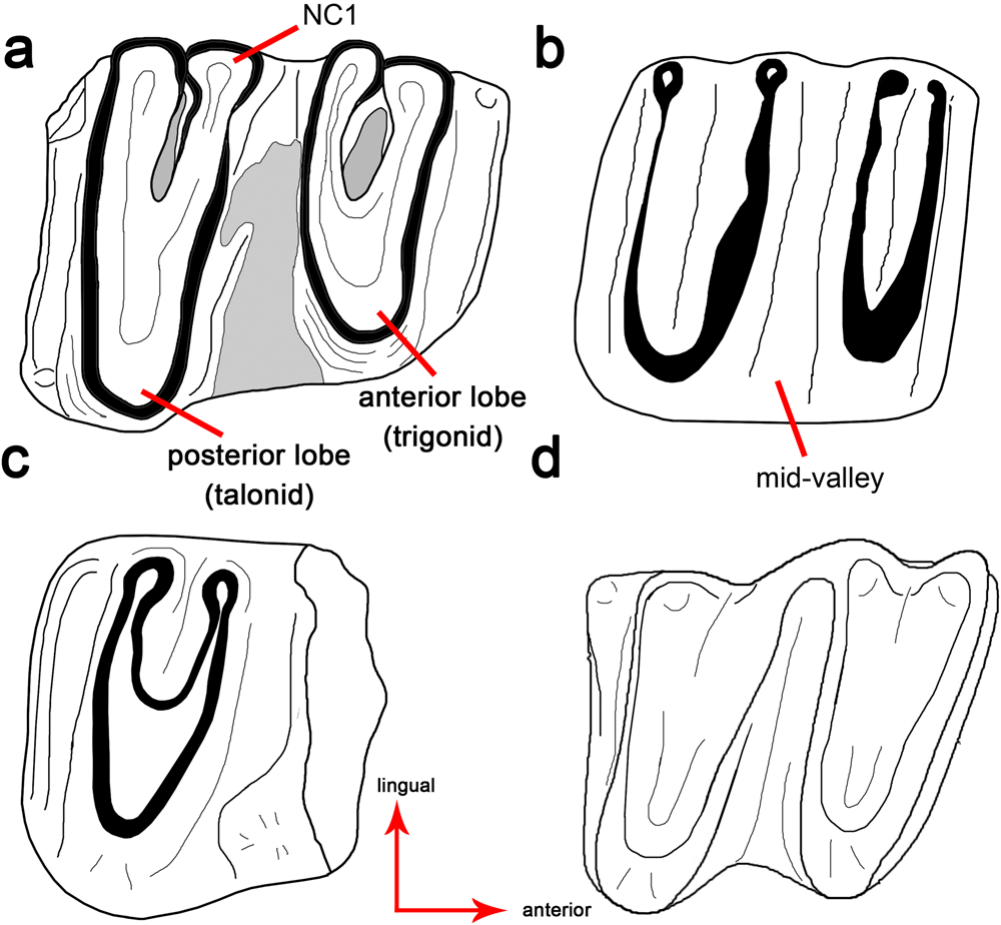
Comparisons of the second lower molar of selected monotremaformes in occlusal view. **a**, *Patagorhynchus pascuali* (based in MPM-PV-23087); **b**, *Obdurodon insignis*^2,19^; **c**, *Monotrematum sudamericanum*^17^; **d**, *Teinolophus trusleri*^20^. Not to scale. Abbreviations: NC1, neoformation cusp 1.

*Patagorhynchus pascuali* sp. nov. (Figs. 1, 2a and Supplementary Fig. 3).

*Patagorhynchus pascuali* sp. nov.

### Etymology

Species name honors the Argentine paleomammalogist Rosendo Pascual (1923–2012), who described the first Cenozoic monotreme remains from Patagonia, thus demonstrating the presence of this clade outside Australia.

### Holotype

MPM-PV-23087, Museo Padre Molina (Rio Gallegos, Santa Cruz, Argentina), a right lower m2 attached to a fragment of dentary. Collected by N. R. Chimento during a joint Argentine-Japanese field trip in March 2022.

### Diagnosis

The same as for genus by monotypy.

### Type locality and age

La Anita farm, Santa Cruz Province, Patagonia, Argentina. The tooth was collected from the “Puma Cave” fossil site (S 50 30.639 W 72 33.617), Chorrillo Formation, early Maastrichtian^7,8^. This new discovery expands the list of Late Cretaceous mammaliaforms recorded in the Chorrillo Formation and equivalent Dorotea Formation in southern Chile, previously known to include gondwanatherians (*Magallanodon*) and dryolestoids (*Orretherium*)^9–11,13^.

### Description

Despite the occlusal surface being somewhat damaged, the morphology of the main cusps and anatomical details can be clearly discerned. The tooth is identified as a second lower molar based on the similarities with the m2 of *Obdurodon*, including a subrectangular-shaped outline in occlusal view, the presence of two lobes each bearing three cusps, a mid-valley lacking cusps, and prominent anterior and posterior cingulids (Fig. 1). Immediately anterior to m2, the fragmentary mandible shows a partially preserved and relatively small alveolus on the labial margin, which presumably corresponds to one of the roots of m1.

The *Patagorhynchus* m2 exhibits a distinct morphology that easily identifies it as a monotreme. This includes a unique lophid and cusp structure resulting in the presence of two mesiodistally compressed lobes that are sub-equally in shaped and size each consisting of three cusps, twinned paraconid and metaconid, wrapping cingulid, hypsodont lobes, and un-basined talonid^12,14,15^.

The m2 is 5.8 mm in mesiodistal length (see Supplementary Results 3), indicating that this tooth of *Patagorhynchus* was possibly intermediate in size between *Monotrematum* and some species of *Obdurodon*. The m2 is mesiodistally longer than transversely wide, and narrows mesially. Six large cusps are present: protoconid, paraconid, metaconid, hypoconid, hypoconulid, and NC1 (neomorphic cuspid 1)^16^. These cusps are relatively low and mound-like and connected by lophids, which form two main lobes or triakididrepanids (Fig. 1, Supplementary Fig. 2).

The anterior lobe (equivalent to trigonid) is labiolingually narrower and apicobasally taller than the posterior lobe (equivalent to a talonid), a condition shared with *Obdurodon*^12^. In *Patagorhynchus*, the anterior lobe is heart-shaped, with the anterior and posterior lophids being slightly convex posteriorly. This results in the paraconids being located anteriorly at the same level as the protoconid. In *Obdurodon*, by contrast, the anterior lophid is anteriorly convex and the posterior one is straight, resulting in metaconid and protoconid being located at the same level. In *Patagorhynchus*, the paraconid is larger than the metaconid, and its base is ventrally positioned relative to the base of both the metaconid and protoconid, suggesting that the paraconid was more ventrally located than the other cusps.

The posterior (talonid) lobe is similar in shape to the anterior (trigonid) lobe, but much wider labiolingually. The lingual cusps are notably taller than the labial one (hypoconid). The preserved bases of the NC1 and hypoconulid are subequal in size and position. The hypoconid is mesiodistally narrower than the protoconid.

Between the lingual cusps of paired lobes there is a narrow, eye-shaped enamel invagination, reminiscent of a flexid. Such a condition is also present in *Monotrematum*^17^ and some specimens of *Obdurodon*^18^. *Patagorhynchus* resembles *Monotrematum* in that the invaginations are delimited by a narrow enamel layer (Fig. 2, Supplementary Fig. 2), in contrast to *Obdurodon* in which the invaginations are labiolingually extended^19^.

Both lobes are separated by a wide, deep mid-valley, which extends from the labial through the lingual edges of the tooth. The margins of the valley widen slightly towards the labial edge of the tooth. The valley lacks well-defined cusps or fossettes, and becomes deeper towards its labial margin.

Posterior and anterior cingulids are prominent, being wider than those in *Teinolophos* but narrower than those in *Obdurodon*^16,20^. The posterior cingulum is eroded on its lingual end, but the preserved segment maintains a constant width along its length, being similar in this morphology to that in *Obdurodon*. In contrast, the cingulids become lingually wider in *Teinolophos*^20^. The anterior cingulid hosts a small cusp on its lingual end, whereas in the posterior cingulid the labial end shows a cusp (the lingual end of this cingulid is eroded, precluding the recognition of cusps), similar to the morphology in *Monotrematum*, but differing from that in *Obdurodon* (Fig. 2, Supplementary Fig. 2).

The tooth bears two roots that are broad labiolingually and constricted at mid-height; they are obliquely oriented with respect to the main axis of the tooth. Regarding the root number, *Patagorhynchus* retains the ancestral condition in m2 shared with *Teinolophos* (and probably *Monotrematum*), differing from the multiple roots present in *Obdurodon* and *Ornithorhynchus*^16,20^.

## Discussion

**Fig. 3:**
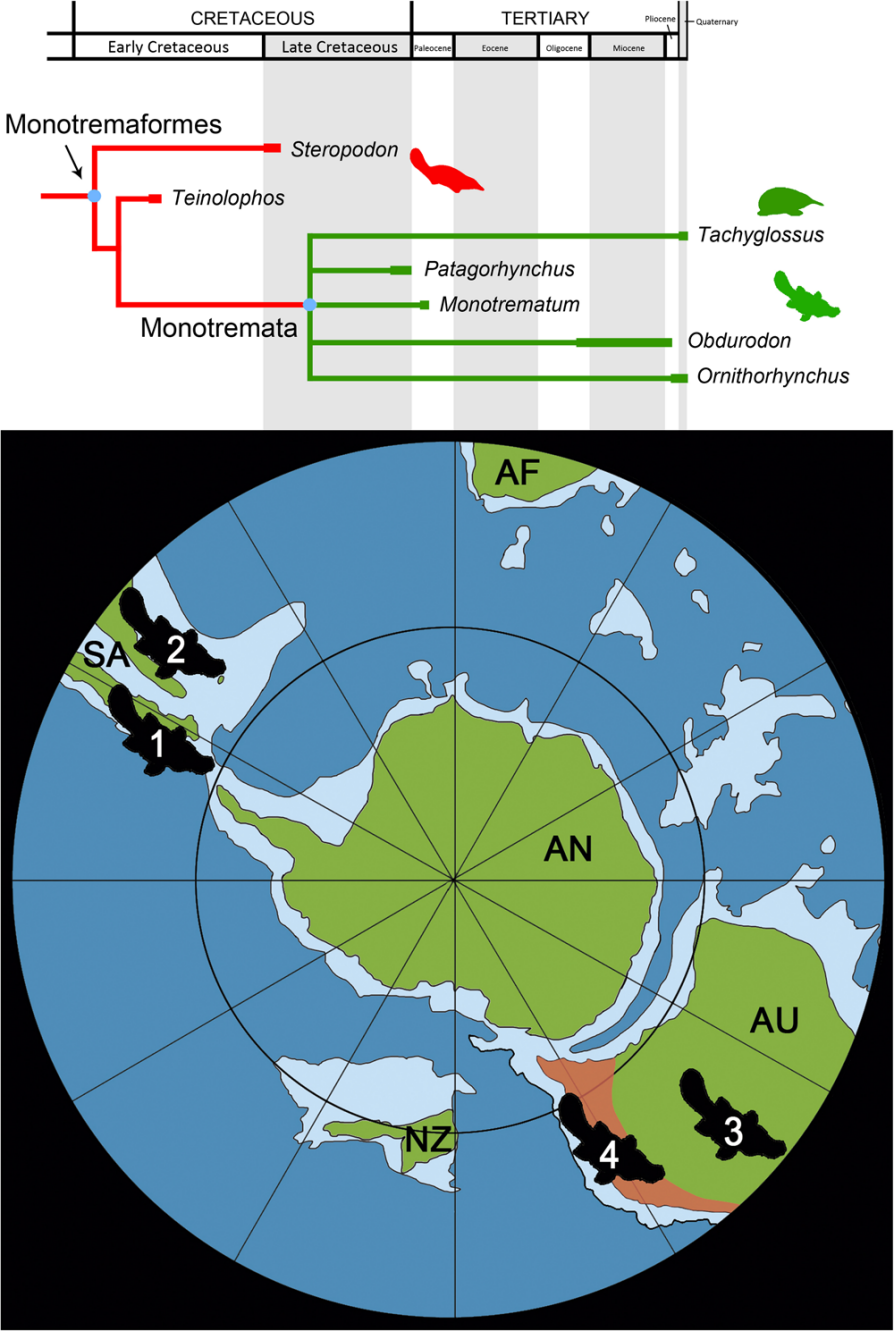
Simplified calibrated cladogram showing the phylogenetic affinities of *Patagorhynchus pascuali*. Basal Monotremaformes^44^ are indicated in red and Monotremata in green. The Late Cretaceous (Maastrichtian) palaeogeographical map (based in Scotese^35^) indicates the fossiliferous sites that yielded fossil toothed monotremes and distribution of the extant platypus *Ornithorhynchus anatinus* shaded in light brown. [1], occurrence of *Patagorhynchus pascuali*, La Anita farm, Chorrillo Formation (Maastrichtian, Late Cretaceous); [2], occurrence of *Monotrematum sudamericanum*, Punta Peligro locality, Salamanca Formation (Danian, lower Paleocene); [3], occurrence of *Obdurodon* spp., different localities from South Australia, Queensland, and New South Wales Oligocene-Pliocene); [4], Pleistocene occurrences and geographic distribution of extant *Ornithorhynchus anatinus*.

As indicated above, the crown shape of *Patagorhynchus* unambiguously indicates that this taxon belongs to monotremes. With the aim to test the phylogenetic position of *Patagorhynchus*, we scored this tooth into a previously published data matrix composed by 558 characters and 128 taxa^21^ (see Supplementary Methods 1 and 2). We concentrated on the characters available in this new tooth, a total of 54 characters can be scored for *Patagorhynchus* (Supplementary Results 1 and 2). The results of the phylogenetic analysis consistently place *Patagorhynchus* as nested within monotremes, together with the genera *Ornithorhynchus, Tachyglossus, Monotrematum* and *Obdurodon* (Fig. 3, Supplementary Fig. 1).

Australia has yielded the most complete fossil record of monotremes^2^, including an array of Barremian through Cenomanian taxa, as well as several species of the Oligocene-Pliocene monotreme *Obdurodon*. In this context, the presence of the toothed monotreme *Monotrematum* in the early Paleocene of Patagonia^1,22^ was interpreted as the result of a single dispersal of monotremes from Australia to South America, before or during the Late Cretaceous or early Paleocene^2–6^. Discovery of *Patagorhynchus* clearly demonstrates that the monotremes had already attained a wide paleogeographic distribution, stretching across southern South America, Australia, and Antarctica, the later one as a connecting pathway (but fossil monotremes are still unknown from this landmass), constituting a clade characteristic of the Weddelian Paleobiogeographical Province^23–28^.

The new discovery expands the list of mammals documented in the Chorrillo and equivalent Dorotea formations of southern South America, adding the Monotremata to the assemblage of non-therian mammals (i.e., gondwanatherians and meridiolestidan dryolestoids^9–11,13^). Remarkably, monotremes are absent from the extensively sampled Late Cretaceous localities of northern and central Patagonia^2,29,30^. Such a difference among mammalian assemblages characteristic of Patagonia is consistent with the uneven distribution of non-avian dinosaurs in this region. For example, megaraptorid theropods, colossosaurian titanosaurs, and elasmarian iguanodontians are numerically dominant in the Chorrillo Formation^8,31^ whereas abelisaurid theropods and saltasaurine titanosaurs are prevalent in coeval units in northern Patagonia. Similar differences are documented in terrestrial and marine biotas between southern and northern Patagonia^32–34^. Thus, evidence at hand suggests that the Maastrichtian vertebrate fauna in southern Patagonia was different from that in northern Patagonia. It is noteworthy that the former had, instead, several taxa in common with Australia (*e.g*., Monotremata, Megaraptoridae). It is likely that a latitudinal zonation of environmental conditions (i.e., dry and warm in northern Patagonia versus humid and cold in southern Patagonia) controlled the distribution and partial abundance of the above-mentioned vertebrate clades.

The presence of monotremes in the southern La Anita fossil site (which occupied a paleolatitude of approximately 60° S during the Maastrichtian, roughly the same as that of southern Australia^35^) is congruent with the interpretation by Flannery et al.^2^ that monotremes evolved under humid, cool and densely forested environments in circumpolar Gondwana. Some authors already proposed that certain anatomical and physiological characteristics of living monotremes (e.g., low metabolism, a mechanoreceptive and electroreceptive beak for probe feeding, and relatively large body size) may have evolved in the context of polar environments^2,18,36^.

The crown morphology of the only available molar of *Patagorhynchus* is closely similar to that of the Paleogene *Monotrematum* and the Neogene *Obdurodon*, revealing a highly conservative dental morphology for toothed monotremes^15^. Remarkably, this molar pattern underwent only minor changes for approximately 60 million years from the Late Cretaceous through to Miocene times. This duration of stasis in dental morphology considerably exceeds that seen in other mammalian groups (*e.g*., therians and dryolestoids^37–40^).

The labiolingually broad segment of the molar of *Patagorhynchus* and the reduction in the number of teeth (eventually restricted to only two upper molars inferred for *Monotrematum*^2^) may be congruent with the duck-billed morphology of the snout documented in more derived ornithorhynchids. In addition, the presence of a hypertrophied mandibular canal in *Teinolophos* suggests the development of electroreception occurred early in the evolutionary history of Monotremata and that the acquisition of a specialized duckbill for high-resolution aquatic electroreception is unique to the clade^39^. Based on such evidence, we hypothesize that a highly sensitive duck-billed snout is likely to have already been present in Late Cretaceous monotremes, such as *Patagorhynchus*. Apparently, a similar anatomical inference could be made for the rest of the body, as suggested by the morphology of the distal femur of *Monotrematum*^41^ being almost identical to that of the living platypus. As in *Ornithorhynchus*, extinct monotremes may have had a sprawling posture of their hind limbs, and eventually adapted for swimming^42^. The possibility that *Patagorhynchus* had already acquired ecological and behavioral characteristics similar to those of the living platypus, which inhabits ponds and lakes, is congruent with sedimentological evidence suggesting that such environments were prevalent during deposition of the Chorrillo Formation^7^, as well as with occurrences of Nymphaeaceae aquatic plants, freshwater snails and abundant larvae of chironomid insects, with the latter two invertebrates constituting part of the food for the living platypuses^8,36,43^.

Discovery of *Patagorhynchus* gives an insight into the degree of continuity between the terrestrial vertebrate faunas of western and eastern Gondwana during the Late Cretaceous, suggesting the lack of paleobiogeographic barriers to their dispersal prior to the deep-water opening of the Drake Passage and the Tasman Gateway. The diversification of monotremes towards the end of the Mesozoic suggested by the present discovery implies that an extensive and still unknown history of this clade of peculiar mammals awaits to be documented in Mesozoic beds in southern South America.

## Methods

The material reported in this publication was collected from the Chorrillo Formation (Upper Cretaceous, lower Maastrichtian^7^) cropping out in La Anita fossil site, SW Santa Cruz Province, Patagonia, Argentina. The specimen was found in association with both terrestrial and aquatic mollusks, calyptocephalellid anurans, chelid turtles, snakes, ornithopods, sauropods, and non-avian and avian theropod remains^7,8^. With regard to mammals, the same outcrop yielded remains of the gondwanatherian *Magallanodon baikashkenke* and isolated caudal vertebrae of yet unidentified mammals^8,9,13^.

Cusp nomenclature of molariforms used in the description and codifications of *Monotrematum* and *Patagorhynchus* follows the terminology applied by Kielan-Jaworowska et al.^45^, Rich et al.^46^, and Woodburne^16^.

### Nomenclatural acts

This published work and the nomenclatural acts it contains have been registered in ZooBank, the proposed online registration system for the International Code of Zoological Nomenclature (ICZN). The ZooBank LSIDs (Life Science Identifiers) can be resolved and the associated information viewed through any standard web browser by appending the LSID to the prefix “http://zoobank.org/”. The LSID for this publication is: 01EF7079-F4F8-4996-ABD3-D61BBD04A2BA.

### Reporting summary

Further information on research design is available in the Nature Portfolio Reporting Summary linked to this article.

## Supplementary information

Supplementary Information

Reporting Summary

## Data Availability

The datasets analyzed during the current study are included in this published article (and its Supplementary Information file).
