# Supplementary material for: First monotreme from the Late Cretaceous of South America
Source: Commun Biol. 2023 Feb 16;6:146. doi: 10.1038/s42003-023-04498-7 (PMC9935847; doi:10.1038/s42003-023-04498-7)
Supplement: Supplementary file 1 — Supplementary Information [file 42003_2023_4498_MOESM1_ESM.pdf]

SUPPLEMENTARY INFORMATION

First monotreme from the Late Cretaceous of South America

Nicolás R. Chimento, Federico L. Agnolín, Makoto Manabe, Takanobu Tsuihiji, Thomas H. Rich, Patricia Vickers-Rich & Fernando E. Novas

Correspondence to: Nicolás Chimento, nicochimento@hotmail.com

This PDF file includes:

Supplementary Methods 1. Phylogenetic analysis

Supplementary Methods 2. Characters added to Zhou et al. (1) data matrix

Supplementary Figure 1

Supplementary Figure 2.

Supplementary Figure 3.

Supplementary Results 1. Codifications of Zhou et al. (1) data matrix with the addition of two characters and *Patagorhynchus* and *Monotrematum*

Supplementary Results 2. List of common synapomorphies of the node 172 (Ornithorhynchidae)

Supplementary Results 3. Measurements of *Patagorhynchus pascuali* (Holotype, MPM-PV-23087, right m2).

Supplementary Methods 1. Phylogenetic Analysis

With the aim to analyze the phylogenetic relationships of *Patagorhynchus* we ran a phylogenetic analysis following the comprehensive data matrix constructed by Zhou et al.[1]. To this matrix we added two taxa (*Patagorhynchus pascuali* and *Monotrematum sudamericanum*) and two characters (557 and 558). This resulted in a data matrix of 558 characters and 128 taxa. A total of 54 characters can be scored for the single tooth of *Patagorhynchus* and 44 characters can be scored for *Monotrematum* (known by two upper molars, one-half of a lower molar, and a referred distal part of a femur).

Monotrematum was codified on the basis of the bibliography[2-5] and personal inspection of plaster copy of the holotype housed at MACN-Pv s/n.

Some codifications were made on the original data matrix[1]. These include:

The codification of character 170 regarding the "distal metacristid". Zhou et al.[1] codified this character as 1 (absent) for *Teinolophos*, *Steropodon*, *Obdurodon* y *Ornithorhynchus*. However, we changed the codification for *Teinolophos* and *Steropodon*, following previous authors[6,7], that demonstrate the retention of a distal metacristid in those taxa (Rowe et al.[7]: character 132; Rougier et al.[8]: character 119; Krause et al.[9]: character 434).

Character 193, regarding the number of lower teeth in *Teinolophos* was codified as "?" by Zhou et al.[1]. However, recent works demonstrate that *Teinolophos* had 5 lower molars[10]. In this regard we changed the codification to "1".

Character 218 refers to enamel microstructure. It was codified as "?" by Zhou et al.[1] for *Obdurodon*. Nevertheless, Wood and Rougier[5] analyze enamel microstructure in *Obdurodon* and indicate that the enamel prisms are "normal" (i.e., 2.5-6.5 µm). In this way, the codification of

character 218 is changed from “?” to “2” (full prismatic enamel). The enamel of *Monotrematum*, was codified following Wood and Rougier[5].

The data matrices was analyzed here under equally weighted parsimony using TNT 1.5[11,12]. In both cases, a new technologies search of 50,000 replicates of Wagner trees followed by TBR branch-swapping algorithm (holding 100 trees per replication) was performed. The best trees obtained at the end of the replicates were subjected to a final round of TBR branch swapping. Branches with a maximum possible length of zero among any of the recovered most parsimonious trees were collapsed (rule 3 of ref. 13 and 14). As a measure of branch support, decay indices (=Bremer support) were calculated[15,16], and as a measure of branch stability, a bootstrap resampling analysis[17] was conducted. Both absolute and GC (i.e., difference between the frequency whereby the original group and the most frequent contradictory group are recovered in the pseudoreplications[18]) bootstrap frequencies are reported.

Supplementary Methods 2. Characters added to Zhou et al.[1] data matrix

557- Second lower molar, neomorphic cusp (NC1) anterior to the hypoconulid (applicable only to molars with talonid cusps of triangular arrangement and without entoconid): (0) absent; or (1) present.

Zhou et al.[1] (character 118), codified the entoconid as absent (0) for *Steropodon*, *Teinolophos* y *Obdurodon*, following previous works (Luo et al.[19]: character 70; Luo & Wible[20]: character 87; Luo et al.[21,22]: character 91; Rougier et al.[8]: character 87; Bi et al.[23]: character 117; Huttenlocker et al.[24]: character 118; Krause et al.[9]: character 444), a criterion that is followed here. Further, we endorse the proposal by Woodburne[6], who demonstrates that in monotremes there is no cusp anterolingually located to the hypoconulid. In *Obdurodon, Ornithorhynchus* and *Monotrematum* there exists a cusp located in the same position as expected for an entoconid, but it appears to be not homologue, and thus, is interpreted as a neomorphic structure by Woodburne[6], a criterion followed here.

On the other side, there are some Mesozoic cladotherians (e.g., *Vincelestes*, *Peramus*, *Nanolestes, Kielantherium*, *Atokatheridium*; see Luo & Wible[20]: character 81; Luo et al.[21,22]: character 85; Rougier et al.[8]: character 83; Bi et al.[23]: character 111; Huttenlocker et al.[24]: character 112) that had a complex talonid in occlusal view, that shows several cusps but that invariably lacks an entoconid[25-28], and are codified here as “0” (absent).

558- Lower molars with hypoflexid (mid-valley) between the trigonid and talonid (applicable to the taxa with two V-shaped lobes on each molar): (0) mid-valley incomplete (the distal metacristid joins the lingual side of the trigonid and talonid); or (1) mid-valley complete (both V-shaped lobes are completely separated by a valley without structures).

Supplementary Figure 1. **Strict consensus tree including *Patagorhynchus* and *Monotrematum***. Present analysis resulted in 4800 Most Parsimonious Trees (MPTs) of a length of 2794 steps, and Ci 0.322 and Ri 0.802. *Patagorhynchus* and *Monotrematum* were included in Ornithorhynchidae (node 172).

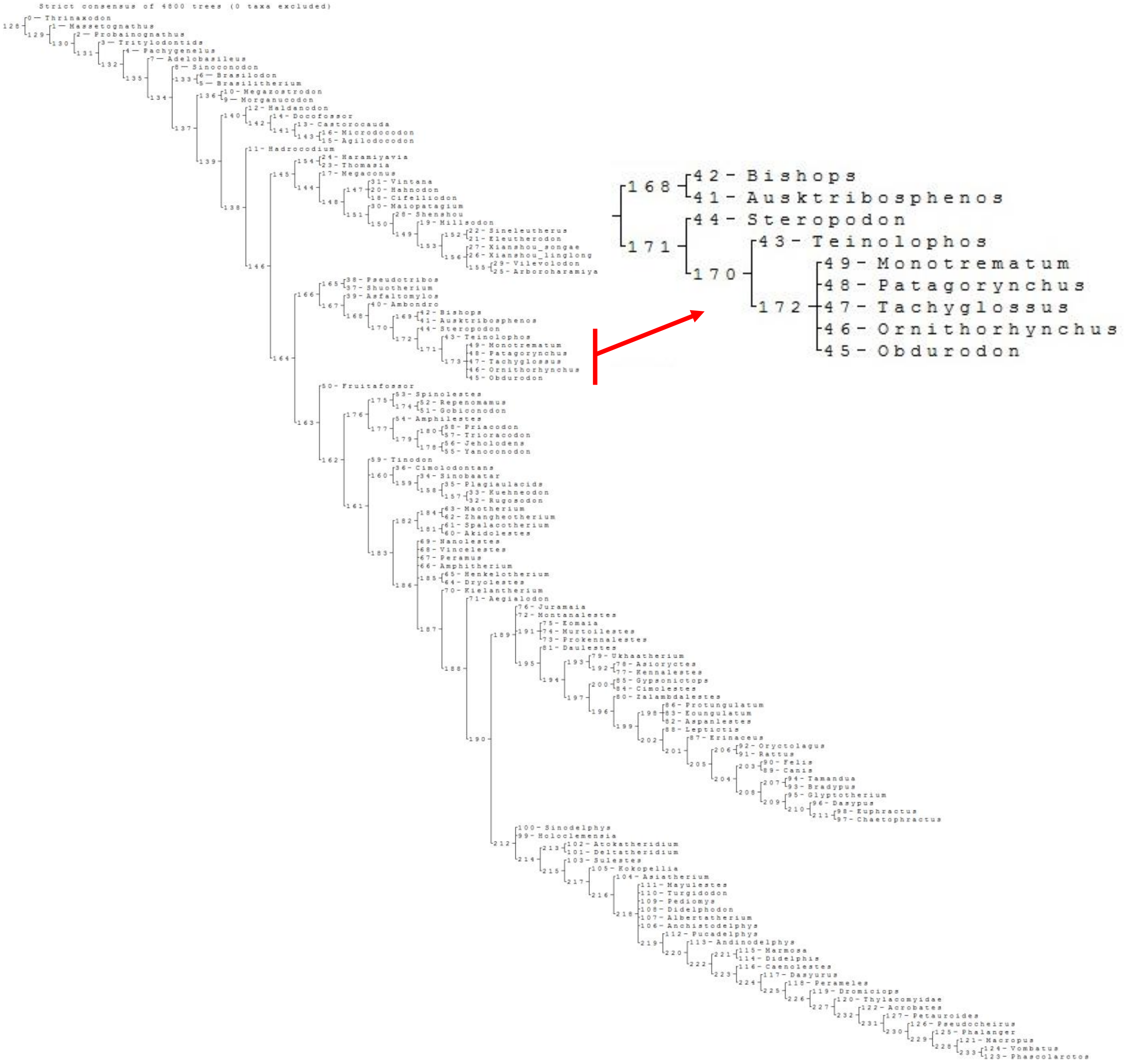

Supplementary Figure 2. **Right lower molars of selected Monotremata**. **a**, *Patagorhynchus pascuali* nov.; **b**, *Obdurodon insignis*[29]; **c**, *Monotrematum sudamericanum*[4]; and **d**, *Teinolophos trusleri*[10]. Abbreviations. ant lophid, anterior lophid; pos lophid, posterior lophid. Numbers refer to newly added characters. Not to scale.

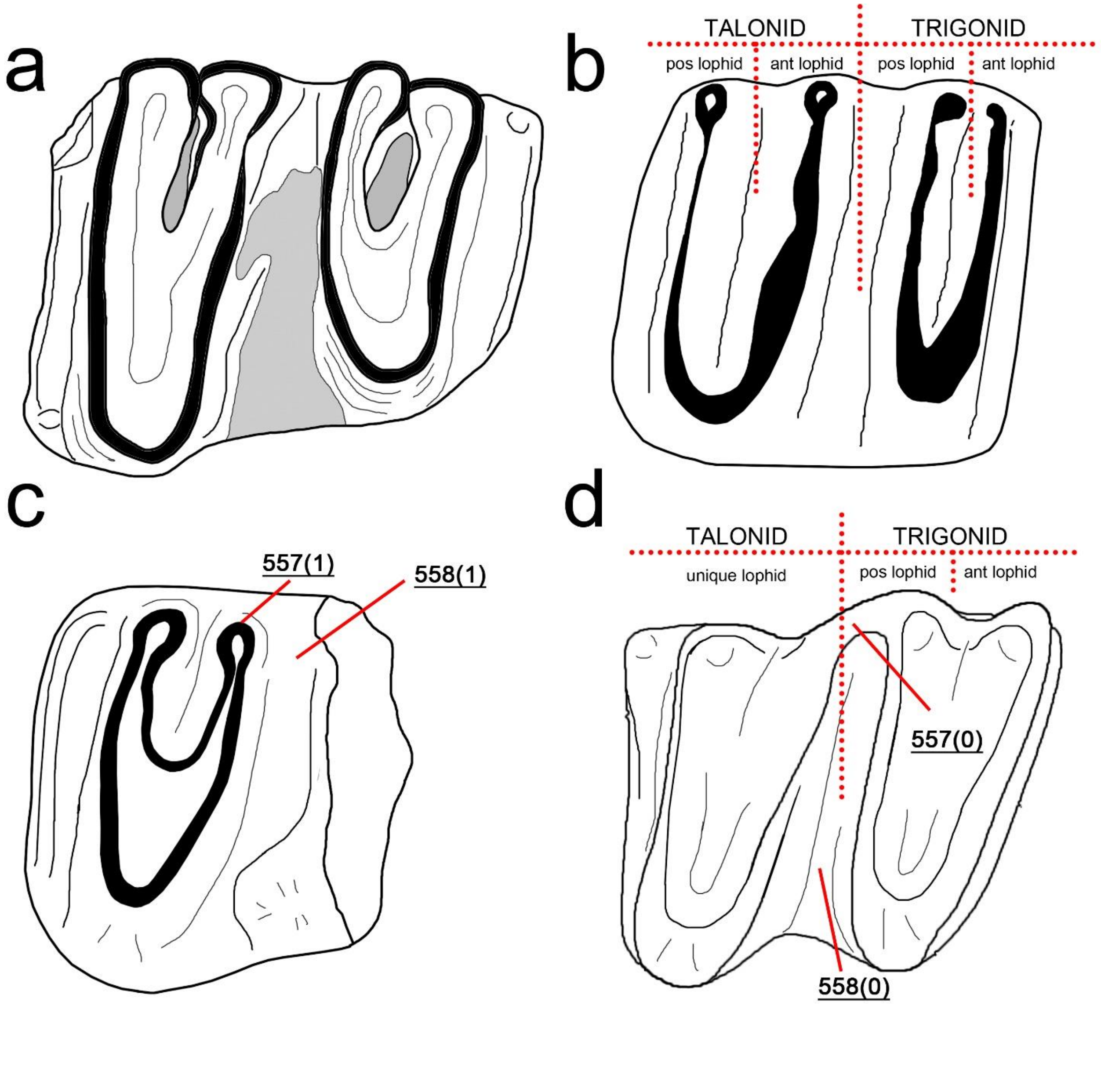

Supplementary Figure 3. Photograph and interpretative drawing of *Patagorhynchus pascuali*, MPM-PV-23087. Lower molar 2 and part of the right jaw, in A, occlusal view; B, medial/lingual view; C, lateral/labial view; D, posterior view; E, anterior view. Scale bar: length 2 mm.

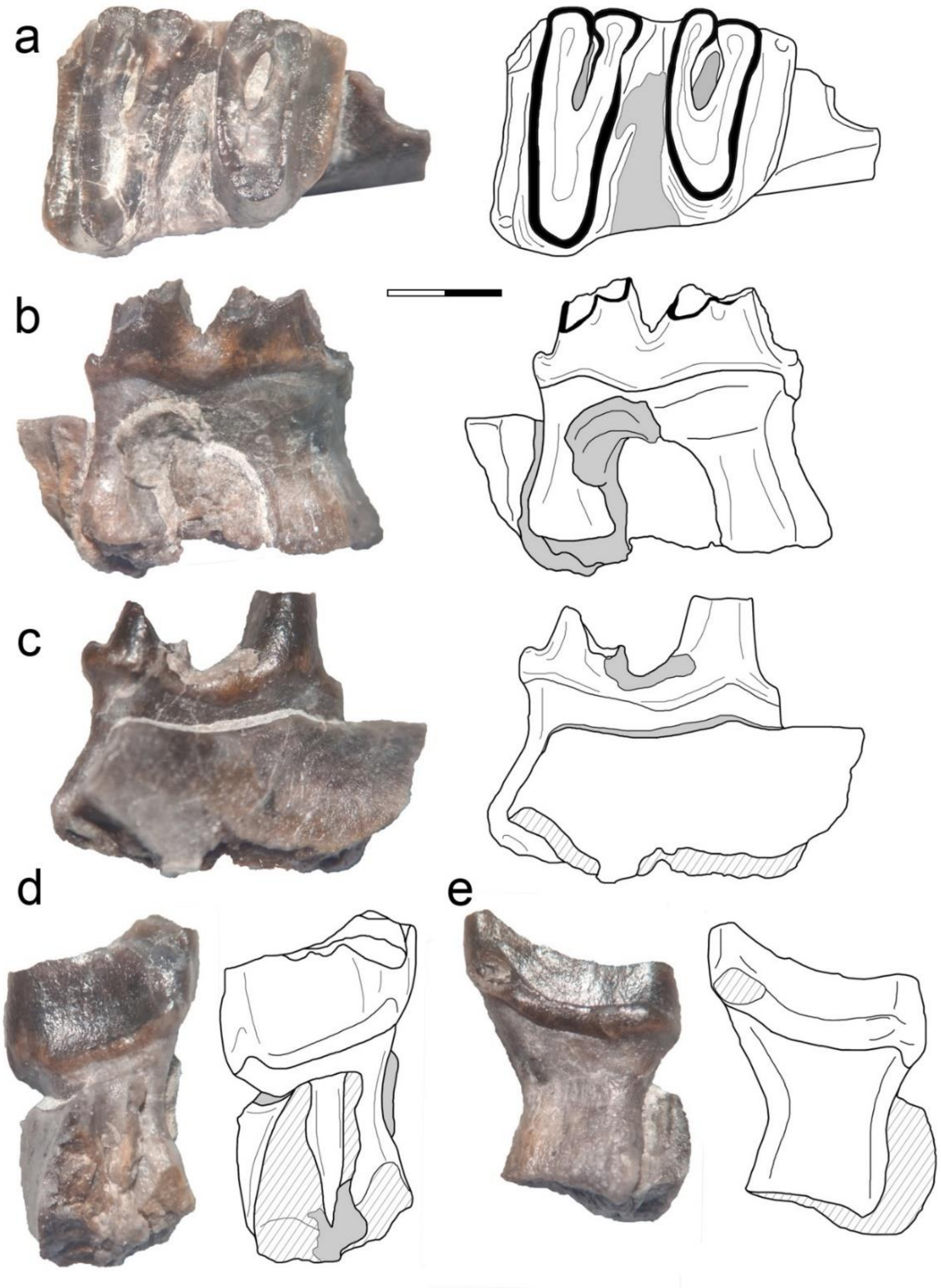

Supplementary Results 1. Codifications of Zhou et al.[1] data matrix with the addition of two characters and *Patagorhynchus* and *Monotrematum*.

Thrinaxodon
000000000000000000000000000?0?0?010000????????????0?0?000000000?????????00????0
000??????????0000?000???0?????????????????00??????????????????????????0???00????
00???? ?????????0000000000000000????00?0?0000001???????????
0000000000001000000000000?0?00 0000?0000000000000000000000????000000??
000000000000000000?0000?00000000000000 00?000000000110000000???000000000?
00????????0000??000??00?0?00?000?0000010?10000 00???0????0?0000?00??0?0?
000000000000000000000000000000000000000000000000?0
000000000000001000000000000?????03000?00000000000?000??00????0?00?????????
Massetognathus
00000100000000000100000000?0?0?00010000????????????0???200000000?????????20????0
400??????????0000?000?????????????????????00???????????????????????????033300????
11???? ??????????00000000000000????00?0?000000100?0???0?0?00000?0???0??00000000??
0?0????? ?0?????00????00000000000000?????1000?0??00000???0??0???0?
00??????????????????????????? ?????????????????????0???0??00?00????????1000??000??00?0?00?
000?000001001000000???0?? ??0?0000?00??0?0?
10100000000000000100000000000003000000000000000001000000?000000000 0?
0000000000000010???01120???00100000000?00000?0?????????????????
Probainognathus
0000000000000000001000000000?000?00010000????????????0???000000000?????????00????0
000??????????0000?000???0?????????????????00???????????????????????????0???00????
00???? ?????????0000000000000000????00?0?000000????????????00000?????0??01000000??0?
0?0??? ?0?????00??????????0????0??????10???0??000?0???0??0???0?
00????????????????????????????? ??????????????????????0??00?00?????0??1000??000??00?0?00?
000?000001001100000???0????0?0000?00??0?0?001000000?
000000100000000000000000000000000001000000?0000000000?000000000000000101?????
03000?000??000??0?000???0?????????????????
Tritylodontids
00000100000000000000000010?000?30002000????????????10??20?000000????????021????2
4000?00??????0000?000??????????????????????00?0?0??????????????????????????033300??
0023??? ????????????2?0300002??2????0000?0?10??11200100000010000100000000?
010000010000000 000000000000000000000[01]1000000?????2000010?
000000000000000000?0000?00?000000 0000000000000000001??000?0?00?000000000?00?
0???0?01111??1000000?00000000?0000010 00000100???000000?0000000?0100?
00210000000000000100000000[01]010301000000000000[0 1]02001000?
1000000000000[01]00000100?0111???00??0[01]1000100000??0?0100000??????? ??????????
Pachygenelus
0000000000000100000000000000010000?20000100?????0??????1?1?000100000?????????00????0
000??????????0000?000??000????????????????00???????????????????????????0???00????
00???? ?????????03003000010000??0000?1?2000001???????????000000?00??0??10???0?
000100?00 ?000?000000000000000100000001000?210001????00?0000000?????
00??????????????????????? ??????????????????????????0000000100?0?0?01111??1?00000?
0000?000?000001011000000???000000?0000010000?1?
002100110000000010000000010000010101010000000001000000?10000
0000000000000000????????????????0?0??00???0?010?????????????????????

Brasilitherium
00?000000?00???00???01100???0010000101000 0??????????0?0100000020???0??????00????0?
00????????????????000???00???00??????????00??00??????????????????????000000??????????
????????0100?0000100100??????????00?00??0?????????????10?????????????????????????????
???
?????????????????0?00?0??0?????????1????????????????????101???????00????????????????????
????????????????????????????100100??0101010100000001000?00??00000?0???0000?000?0??
?????????0?000?0?0??0000000010?????????????????????
Brasilodon
0??000000?0?????0???011000??0010000101000 0?????????00??100000020???0??????00????0?
00???????????2????000???00???00??????????00??00??????????????????????000000??????????
????????01002000000000????00?0??00?00??0?????????????10?????????????????????????????
?????????????????0???0??
?????????????????0?00?0??0?????????1??????????????????????0????????00??????????????????1
?001010?????????????????????100100??01010?0100?000010?0?00?20000000???0000?00??0?
??????????0?0???0?0??000??0?010?????????????????????
Adelobasileus
???0??????000???
???????0000?000??000???00??????????
????0???0???
?????????????????????????????0???
????????00000?????????0221100?????0000001?11??00000001001000 0????000000000000010000
?10?????????0?????????????0??0?0?000?????????00??0200???????0001000????????????0???????
??????0???0?0????0??????0?????????????????????
Sinoconodon
000000100000000000000000001101000000001000??00??????00?11000110000?00?0????00????
0000??????20??01000000?1000???0000???0????00?0?0???00?????????0????????000000????00?
0?0??????000000000000000000003311220200000001???????????100100?0???0?01??????001000
100000000000000000000000001000000???????????????????000?00?0???00???????????????????????
???????????????????????????00000001000 0?000221100100000000001011101010000000000100
0?000000000000001000101010210011000000000100000100101000101000100000000200010002 0
000000010000000000100001 01?????03000?0?0??000??0?000???0?????????????????
Morganucodon
00001010000000000000000000002[01]010000000100 0??00?0000001?01000110200?00?0????00
?0??1100??????20??2100000110000???0000???0????00?410???00?????????0????????00000100
??1100?0??????0000[01]00[01]000000000000[23][12]000000001101???????????000[12]00000
000??11?1?000??1???00?100?00000?0000000001000000?????2100010???00001000 0010?0000
??000?0000000000000000000000000???0?????????0??00000010100[01][01]?00023111120100
000000101110101000110100111 0?01000000000000100010100021002100000000010000010010 1
0001010101000000020001000200001000110000000010000121?????13000?000??000??00000?
?0???????????????????
Megazostrodon
00001000000000000000000000200200100001000??00?0000?01?000001102 00?00?0?0??00?0?
?1200??????20??2100000100000???0000???0????00?4?0???00?????????0????????00000100??1
100?0??????00001001000000000000011000000001001????????????20?200?00000?01101000???
????0??100000000?00000000010 0000??0??02100010?000000100000?000000?0000?00?0000?

00000?00??0???0?0?1?000000000?000000?0010100???0002311012010?00000010111010000011?1001110??1000000000001000101?00210?21???0??001?000010?1??00010101010000??2?0010?0200001?00???????00??0??????????????????0???????????0?0??????????????????????

Hadrocodium

0101?130010?4021100000000200100200?01000??00?0000101?10000110000?0??0????00?0??1200??????20???0000000?0000???0000???0????0????????00????????0????????00000100??1100?0??????00000000000000100033444300000001101????????????0020???0??10000020101?0002311123010000000001?111010?10011021010110010000000000100?1010??????21?0??????1????02012?1030101000100000012?2?10?020101211001?00??000?2??1221???????000?000???0010000?0???????????????????

Haldanodon

000001100010001000100000002101001000010000??00?0000000?01000110201010100?0??00?0??1100??????20??21031200?0000???0000???0????00?1?200?00????????0????????03320121??1100?0??????00000001000000101022000000000001100???????????00020?????????????????????????100101100?00?0000012000010?????21?0???????000?00000?010?0??0???00000102001 1?000231100301000000001012100000?001102101110?0100000000000001001101000????21000???00??00002011?10001010101000000002000100020100111011000000000???1???????010?0000000000??0?0?0????????????????????

Castorocauda

00?00110001???1?000000000002101001000110000??00?0???0???11000110201010000???00?0??1100??????2???20031200?0000???0000???0??????????????????????????????????00?0?1???1100?0??????0000???100?????110?00??0?10??01100????????????0020????????00010000??????????????????????????000012000010000000210000000001000000000101100000?0100?01?110110000000000000000??00?0??0?00000?01100???010??111001000000?????????0??????10??00??2????0??2????1???11?00???????????????????????????????????????0????0???????????????????

Docofossor

00000100000100110?00000000021010030001100000?00?00??00??11000110201010000???00?0??1100?????020??21031200?0000???0000???0????00?1020000????????10????????03320121??1100?0??????0000100100000001010223231100000011000???????????0020????????010?0000?????????????????????????????00001001011?21000?0?0010?00000001011001101000?0101101100000000000000000001000000000100001001????1???00??????????????????????1000??????????????????????00???????????0??????0??????????????????

Agilodocodon

000001100011001100000000000210100200011000000?00?000000??1200011021112010????00?0??1100?????000??21031200?0000???0000???0????00?1020000????????10????????03320121??1100?0??????00001001000000111100220011000011000???????????002000?????1010?00000??????1101001????0???0?00?01100111100010210000000001000?000001011000?0100?01011011000000000000000000100000001100001001????1020011??0?231100?????0000001?12?????????1??2101110????0000000???1001101000210?21???1110010000?2??1???00??????100??????????0002??0??????1000????????????????????????00???????????0?0?0??0??????????????????

Microdocodon

0000011000110011 0?0?0100002101002000110000?00?000000??1200011021112010????00?0??1100?????000??21031200?0000???0000???0????00?1020000????????10????????03320121??1

100?0??????0000???100?????111002200??0?????0?0????????????020?????001010?000000000
?111010011?1000000?001011001110?????21000000?0100000000101100110100??1011?1100
000000000000??0?1000000?000?0?00[01]??001?2??11????23110????????000???????????????
????????????00????????????????0?????1???1110010000????1????0???????0???????????0??2??
??1???0???0????101100000????
Megaconus
10000?100000001?000000000?220100?0002100100000?????011??2?0112201??300000221????
240100011?????0??00?0122???033401000013??
????????????301400??2??2??333343000?2???12?0000011101?10300?00?0010111000000000???
11?0?0??????00?00000?00001000????021?0??0000???0?00000?200210?00?0??0000?00000000
0???00?0??0???00?0??????00100????1???
???????????????????00?????1???011??10000??????????????????0???????????????????????????
????????????????00???0??????????????????0???????????????????
Cifelliodon
??????????????????????????2??10???1???????????
???0????0?????1?????????????
??31??????1?0???????4????2?????????????2??0???030??????????????????????????????????????
???
????????0000101?111?00?231100??????????01?11?0000??01???11??????1000000??0001?0?10
10??????????????????????00000??3011300010?001012??0?0?221??000100210110000?000112
1????012011111111111100111111?0???????????????????
Millsodon
??111??1???????????
???0??3?0???013????????????
?????????????????????????????????????0??0????3???
???
???
??0??????
????????????????0???????????????????
Hahnodon
???10???1???????????
???0????0???013????????????
?????????????????????????????????????2??0????3???
???
???
??0??????
????????????????0???????????????????
Eleutherodon
???????????????????????????????????????1????0??????????????120???????11122?????24112
1120?????0??0??0120???0443010?0113????????
???????1?????????????????????0???????01111001?01?103???????????????????????????????????
???
???
???00
??????????????????????1???????????????????

Sineleutherus
???????????????????????????????????????111?1??????21??2?0?120?0???01????21????24112
01[02]0?????0??0??0120???044?010?0113?????
??????????1???????????????????????????011110?1?01?103???????????????????????????????
???
???
???
00??????????????????????1???????????????????
Thomasia
??00001?????0?0??0??00?000???10100021????2401
10100?????0??0??0??03330???0013???????
???????????000???????3323????0?3???1?1?1010??000??030???????????????????????????????
???
???
??1
1??????????????????????0???????????????????
Haramiyavia
0?0001000000000?0?0?0000000100000000100?????????????0??0?0000000??200100021????
240110100?????0??0??0120??033010?0013??
????????????10010000??0100?133?1??0000??1210101000000?00?0????????????????????????
???
???
???00?????????????2?????????1?????????????
??????11????????????????0????10???????????????????
Arboroharamiya
1111?13000012021?0111100002203001000210111210??????21??233001000??3??210221????
241121120?????0??0??01?2??0334000?1113??
????????????4??400?02??2??334443??023???120111100?001?103?????????01??10???????0?01
?????????????????????????100??12[01]??0???00??0?00???100??00?00?????????????0??????
?????????????0???001?0?01???
??????????????????????????1110?1???
????????????00?????????????????????11???????????????????
Xianshou_linglong
111??13000012021?0112100002203001000210111210????021??233001000??3??111021????
241120100?????0??0??01?0??043400??1113??
????????????301400002??2??334443??023???120111100?001?103???11??101??100?????????
????????????????000110000?10010012100201?0000???0000??00?000?00??????00?0?0001000
0?0000?000000000000100100?01???
??????????????????????????1????????????????????????13???101???0??????????????????????
?????????????????00??011???0???????1???11??????????????????
Xianshou_songae
1112?130000?2021?0112100002203001000210111210????021??233001000??3??111021????
241120100?????0??0??01?0??043400??1113??
???????????????400??2???2??334443??02????120111100?001?103???11??101??1???00???01?
1???????????0???0001100001100??012100201??0?000???000100?000?000?????00?0?0001000
0?0000?0000000000000100100101???

??????????????????????????1???
????????????????00??????????????????0??1110?????????????????
Shenshou
1111?13000012021?01121000022030010002101111100????02[01]??222002000??3??110021?
???241111000?????0??0??01?0??033300??001
3??????????????4??400002??2??334443??023???12011?100?001?103????????10110100?00001
0111000000011000000000110000110010012??0201?00?000?00000100?000?000?0???00?0?00
?10000?0000?0000000000001001001011??0101?110??0?231?01?????????????1??????????????
???????????????????100????????????1??????????????20??????0??????101?010???????0?????0
2?0??1001??????1?????????00???111??0???0???1?0??0010?????????????????
Vilevolodon
1111?130000?2021101111000022030010002101111210????021???33001020?????211221????
241121120?????0?00??00?0???044400??1113??
????????????4??400002??2??3?4443??023001?201111000001?10300?0?1?0101??10000100001
11000000011000000000110000110010012100201?000000?000001000000?000?00000000?000
10000?00000000000000000100100?0?1?00101??10??002?1?01?????????????????????????????
??????????????????10??????00???0?????1110010000????0????00?3010101?011??????0012???
010??0?00??????????????????????11????????????111??1110????101??0?00????
Maiopatagium
??100?????02?????????????????000021????24????
???0???00???013???????????
???4?????002?????3??44????22??1???0?????00???10?00?0?1?01010?100001000011100000001
10000000001100001100100121002 01?000000?000001000000?000?000000?0?000???00?00???
00000?000?00???100?0????0???
??????????????????????????????????20?012??0113010?01?011??????00?2????[12]???0000??0
0?????????????????????1??????10?????0??0010?????????????????
Vintana
???0?5?????
?????????????????????????????????????0??????????????????????????0??300???02????????????
???31??????2?????????24????2??????????????????21020???????????????????????????????????????
??
?????????00000010111?000231111311111101001012 1?000??011121?1111??1000000?010110?
?1?11???????????????????????00121???01110000000011?2120?00220??000??1010?100?11111
02[12]1??????2011?11011111?011111?0???????????????????
Rugosodon
2112?10??00?302?10102120102224001000210110001??????030??2?00001?0????1000121????
240111000?????0??0??00?0??033300??0023??
????????????201400001102??01442200103?11122010010010 0?002011111010?112110000001
10110110011110011001 00?00001011000102 1?011000000?1?001002011001000121041000100
0012001000111000101100011100002101?0???
????????????????????????????????????1???????111000?????????????????0???????????????????
????????????????????????00????1??????????????0??0001????101100000????
Kuehneodon
2112?10??0013021101021201022240010002101100011?????030??2?0000120??311000021???
?240101000?????0??0??00?0??033300??0023?
?????????????211400001102??11443200103?111220000100100?00201??????????????????????

???
???
???1???????
????????00??????????????????0??00???????????????????
Sinobaatar
2112?10??0023021101021201022240010002101100010?????030??2?0000010??201000121???
?240101001?????0??0??00?0??033300??0023?
?????????????210400002??2??024423001?0?11120000001 0112?0020?11?10100112110000001
101101100111100110010020000101100010210[01]11000000011101002011001000121041000
1000012001000111000101100011100002101000010101 10?000????????????????????????????
???????????????????????????????????????1????????111000?????????????????0??????????????
?????????????????????????????00??????????????????0??00?1????101100001???
Plagiaulacids
2112?10??00[12]3021101021201022240010002101100011?????030??2?0000120??211000121?
???2401[01]100[01]?????0??0??00?0??033300?
?0023?????????????211400002??2??0244[23]300103?11120000001011200020?????0????????
??0????????????????1???????????????????????????
??????????????????????????0????????????????????231111301??00000010121110110021 1???1
1110?10?00000000010001111??????21?0??????????0?00?20100110200010 0?0000202?112220
2000100[01]11011001?21????????00????????????????0?01000???????????????????
Cimolodontans
2112?10??0023021101021201022[12]40[01][12]00021011000[01]0?????030??2?0000000??200
000[12]21????2401[01]00[12][01]?????0??0??00?0???
??????033300??0023????????????????3004[01]0002??2??1[23]443300120?[12]1120?0000101122
0020010110100112110000001101101100111100110010 0[12]0000[01]01100??0210111000000
0111?100201100100012104100020211120010011110011011000111000021010000101 0[01]10
?00023111130100[12]00[01]0010121110110021121[01]111[01]0010[01][01]000000001[01]00
1110323121211001 11??21110000[01][12]01001102000100200002[01]21112220200110012101
[01]0[01]0021110221???00[12][01][01]11[01]000?000[01]11010100001??????1??????1???
Shuotherium
0010110??0000021??000?00?0????0?000101000???????????0?11111000011??0?0???13?2001
200?????0100?13230210?01110000100???0???11?1?1002?0000?0??0000000?10011200????2
21100??01001110????????????????3?????0???????????????????002?????????????????????????
???
???
???
???
Pseudotribos
0?10011000?0001??00??????2101010001110 00??????0???00?111110000?1110?0???13?2001
200?????0100?13230210?0111000?100???0???11?1?1002?0000?0??0000000?10011200????2
21100??01001110??01?0??100?0?003310000??01100????????????0020??????101??00??0010
001????????????0????0???12000000?????211???00000?10?0000000?00?????????????????????
?????????????????????????0???0???
???0??????????????2???1??????????
?????????????????????????????????0???????0???????????????0?

Asfaltomylos
0012112110000021??0022000021310120010??00?????????????11011?0????????0????13?2??1
300???????13?02100000??111022141100011112????0??????????????????????011?0?????221
1?1102??00000??????????????????????0???????????????????0?2????????????????????????????
??
??
??
???00
Ambondro
?????1???00????1????????0?????0???0?0??00?????????????12131?0??110???0???13?2??1300
?????0?13300120100??111022241120?11112??????????????????????????011?0????2211?1
1020100000??????????????????????0???????????????????0?2???????????????????????????????
??
??
??
???00
Ausktribosphenos
0010012010000021??002200002??101200101000??00?????????12131000011011?0???13?2??
1300?????0213300120100?0221022141220032123????0????????????????????????033?0?????22
21?11121110000????????????????3????00???????????????????0?2?????????????????????????
??
??
??
???00
Bishops
01?10120100?0021??0022000021310120010?000??00?????????121310000?1011?0???13?2??
1300?????0213300120100?1221022141220032123????0????????????????????????033?0?????22
21?11121110000???????????????03??0?00???????????????????002??????????????????????????
??
??
??
???00
Teinolophos
2100112010023021??0022000?21310120010??00??00???????????????0??????????????3?2??13
00?????1111200120010??222042231210?02?23??????????????????????????????33?0?????2221?
2113002000??????????????????1?????????????????????????0?2??????????????????????????????
??
??
??
??00
Steropodon
011001???0??00?1???????????????????10??????00???????????????0????????0???13?2??1300
?????1111200120110?0222042231210?02?23????????????????????????????033?0????2221?2
113002000??????????????????3??3??????????????????????0?2???????????????????????????????
??
??

???
???00
Obdurodon
21?2?12010023021101122000121311121110?0??00?????????1013100??00????0????13?2??1
300?????1111200120010?0222042231210?02?2320?1????????????????0???????033300????222
1?2113102000?5??5????2??2??333443??0????1?????????????20020?????????????????????????
????????????????????????????????21???1????11???
?????????????????????10000211011 0?000231111????0000001?111?1101001112101110??1000
0000000000?00?0?????????1?0???????????03012?10001?40?110021121202011222?001211001
1?1??00002????2??????0?0??1000?000011010?0???????????????????11
Ornithorhynchus
21?2?1001002302110112200012131112111[02]1000??00????????????????????????????13?2?
?1?00?????1?1?????2?010?0222042231210??2?2320?1???????????????????????033300??????
???2?1?1?2????5??5????2??2??334443??0????1?????????????0????1111011111010000001000
10000001010000000000000120000000100 1?21111100001110000000220110021000 0?0000000010
1100000000010001100001101 00?11[01]010000211001 0?00023111141100000000101110110 1
001112101110?110000000000000?00?0?323121211001110121010030110100012400110 02112
12020112221001211001 1?1?1000021112210 00??010??0000?000011010?0??10000?111210011
0111
Tachyglossus
21?2?10??002402110000000002131111??1?01000??00???????????????????????????????????0
0??
??????5??5????2??2??334443?????????????????????????1111011111010000000100010000001
0100000000000001200000001001?21111101001110000000220110021 0000?00000000101100000 0
00010001100001101 00?11[01]0100002110010?000231111411000000000101110110100111210
1110?11000000000000 10?00?0?323121211001110121010030121102012400 11?0211212021112
2210012110011???[12]0010211122100 0??020???000?010011010?0??10000?1112100 1101??
Patagorynchus
????????????????????????????????????0????????????????????????????????????3?2??1??0???
??0211200020010??222042231210?0??23??????????????????????????0?3???????2221?2113
102000???002???????????????????????????????????
???
???
???
??11
Monotrematum
????????????????????????????????????0????????????????????????????????????3?2??1??0???
?????120????010????????231210?01?2?20?1??????????????????????0?330????222??21131
02000??200?????????????????????????????????????
???
???
???
???????????????????????????????????????11
Fruitafossor
21?001400002002 1??110000112101011001?1000??00?0000000?10?00100000?0??0??????????
1000??????????0??00000?0????????????????00?0????????????????????????55400????0????

?????????????02?0??100100223332000?001100???????????30100????????0100001O???????0??
000011000?001000001200010010010 21??????0000???00??0100?100?0?0????????0000000000
000?0?0000???????????001??0????10???11??
???00?????????????2?1??????????????
??????????????????????????????????0??????????????????????????
Gobiconodon
21?1010??00[12]1021101221001121140010010100 0??00?0000001?11010010010000?0????00?
0??1200?????010??1210000100?00???0000???0????00?110???10?????????0????????000100?2?
11101?0??????00012?03000010010011220000000001010???????????20021?0?1??01010100110
0001101112111111100111000001110001000 0??21002100001000000002110?000?0000?00?00
0010001000000000000000????????0??0010000001120111?00023111130100000000 1?1211200
00011110011111020000001100011 1?1011???????1?1????12????0020021100011100000 10001
120011112212101100 01?0?0001??0??0?00???????0?????1???????0?0?0???1?????????????????
Repenomamus
21?1010??0021021101221001121140010010 1010??00?0000001?11010110010000?0????00?0?
?1200?????010??1210000100?00????000???0????00?110???10?????????0????????000100?2??1
101?0??????000120030000100100112200000000 01010????????????002100110101010100 11000
011011121111111001110000011100 0100????210021000010000000002 1100000?0000?0000000
10001000000000000000?0?0??1?00?0110000001120111?00023111130100000000 1?12112000
00111100111110 20000?011000111 1101???????21?1????12?????020021100011100000 1000112
0011112212101100 01?0?0001010??0100?????00????0?1???????0?0?0??01????1011?000?????
Spinolestes
21?0010??002102110122100112214 01?0?1010000??0?0000000???000100?????0?0????00?0??
1200?????010??000000013??00???0000???0????00?110???1?1???????0????????00000?????11
010???????000120020000100101212232000000000 10????????????0021???11101010?00110?00
110111211111110011101000111000 10????021002100001001?11111110000000000?00000011
00010000000000??0??????0?1????010?10???1120111?000231111?????0000001?12?1??????11
?11111?11020000?011000111110 11??????21?1????12??????2??2???0???????001?????2?????1
22???0?0????0???????????????????
Amphilestes
21?11?0??002102???122??0??211400200101?00??00?????????11010?10000000?0????00?0??1
200?????010??1010000100?00???0000???0????00???????????????????????????000?0?????1201
?0??????0001????????????00??21??0000??????0?????????????002????????????????????????????
???0???
???
???1???
?????????????????????????0????????????????????????
Yanoconodon
21?1010??0021021101221001?2214001000010 00??00?0000001?00000110000000?0????00?0?
?1100?????010??0100000121?00????000???0????00?0?0???10?????????0????????0000120??1
100?0??????000130030000000001333343000100 1110?????????????002000?1000101010000000
0110111211111110011100000 10101000000 1?21002100000000000000001100000?0000?0000000
10001000000000000000?0?0?01??0?0200000001020?11??00?????????????????????????0????
?????????????????????????????32312121???11012210100?00?????0??????110?????2????1022
??0?0????0?????1011?000?????

Jeholodens
21?1010??00?10211012??001?2214001000001000??00?0000001?01010110000000?0????00?0??
1100?????010??0100000121?00???0000???0????00?0?0???10????????0????????00000120??11
00?0??????00011001000010010?33223200000011111????????????002000100?01010200000000
110111211111110011100??01010100000010210021000000000000001100000?0000?0000000?
0001000000000000000000000100000000000000102?011?0002311?130100??0???10??1????????
????????????0??0?0?????????????????21????????1221????????????01?10??110????0?????1022
???011??01?????????1?????????????0?????????????0?0????01????10???00??????
Trioracodon
21?10?0??0021021??1221001022140010000100 0??00?00000?1?01010110000100?0????00?0??
1100?????110??1[02]00000121?00???0000???0????00?1?0???1?????????1????????000000112??
1100?0??????0001??0300??000000123321000??01100????????????0020????????????????????
???
???????????????????????0??????????????02311?????????0??0?0??1010???0????????????????
0??0?0???1??10????????????0???????????0???????0????????1?????????????????0?????????????
??????????????????????????????????0???????????????????????
Priacodon
21?1010??0021021??12210010221400100001000??00?0000001?00010110010100?0????00?0?
?1100?????110??1200000121?00???0000???0????00?1?0???10????????1????????00000112??1
100?0??????0001???300??0?0000122221000??01101???????????10020?????????????????0????
???
??????????????????????0?????0????1??00231111301??000000101?12100??0111100??1000??
000???00??1100101???????2??0???????????020?????001??????10???????[12]??1022?10??????
????0??0??1??????????????????????10?????????????????????????????
Tinodon
2??11?????0[01]1021??121?00???104?130?00??00?????????????01010?00000?00?0????1201??
1200??????1000101000010 0?110000100???00??010?1?1????00???0???0????0?0002210?????2
211?0??????0000???????????1???22??1??0??????????????????0?2????????????????????????
???
???
???
??
Akidolestes
2????10??00?20????1221001021140110011100 0??00?00000?1?11010000000120?1????130201
1200?????010001000000130122 00?0100???00??020?1?1????000??0??0000000?0002220121??
222100??00000000100100??1001100001000000001110????????????0020?????0?0011?000001
00110110211111110011101111101110100001?21111201000011?00000210010210010000000 0
1?00201001000111000010000121000?021010?001020??1???????????????????????????????????
???0??????100?0???2??????12?
???????0???0???????????????????????????????????0?0????01?????????????????
Spalacotherium
2111010??001202?????2100102114011001110 00??00?00000?1?1101000000010 0?1????13120
01200?????01000100000013011200?0100???00??020?1?1????000??0??0000000?0002220121?
?222100??00000000?0?????????100?000?0000000??10????????????0020???????????2????????
???1??????0?????????????????????????
???

??0??????????????????????????????????
??

Zhangheotherium

21?0010??0012020??1221001?211401010111000??00?0000011?11010000001?00?0????13110
01200?????010001000000130111OO?0100???00??020?1?1????000??0??0000000?0002220121?
?222100??00000000200200??1001002200000100001110???????????20020?1110?00011211000
100110110211111110011101111101110100001021112200000001?111002100100?0010000000
0103020100100011100001000002000000 2101????1020111?0012311112????1101001?1?1?10?
??0??1210111100200000000000001?0?11?1??????21??????1??????0??????0??1?????100???????2
????12??2????????00????????????????????????????????????0?0????01????10???00??????

Maotherium

21?0010??0012020??12210010211401010111000??00?0000011?11010000001?00?0????13110
01200?????010001000000130111OO?0100???00??020?1?1????000??0??0000000?0002220121?
?222100??00000000200200??1001012200000100001110????????????0020?11?0000011?11000
?00110110211?1111001110111110111010000102111220000000 1?1?10021[01]?100?00100000
00?103020100????11100001000002[01]0000021010?001020111?0012311112????1101001?1??
?10?100?11000111???2000000000001???11?????????????????12?????0201??20?0??20??100000
????2????12?02?1???0??00??????1??????????????000???0????0??0?0?0??01????101100000????

Dryolestes

21?1011100012020??11110?0121130120011100 0??00?0000011?00000110001?00?0????13010
01300?????0200002000000?01110000220??000??110?001????000??0??0001000?0002220121?
?221100?0010000000100100??00000011000000000301110???????????20020??????????????????
??
????????????????????????????1??
??1???10?0???????????????????????????00??
0???????????????????????????????????0????????????????????????

Henkelotherium

21?1011100012021??111101??2113012001110 00??00?00000?1?000001100?0?00?0????13010
01300?????0200002?00000?01110000220??000??110?001????000??0??0001000?0002220121?
?221100?001000000??0?????00?0?0110000000??0???0?????????????0?20?????0??011?1100??0
???011021111?110?111011?1001110210001?21?0220000000111000021100 00?10?????????1??
?????????????????????????0????0?1011???1020?11?00?23111?3??????????????????????????????
??????0??1?????20001?0??1???????????20??????
???0????0???????????????????

Amphitherium

21?1111100012021??11210?01210301200111000?????11?0?0??00000????01?00?0????130100
1300?????0001002000000?00110000210?0000??1111001????000??0??0001000?00022101????
2211110000000000???100?????0?0?000?0?00????????????????????0?2?????????????????????????
??
??
??
???

Peramus

21?1111100012021??11210101210301200111000??00?1100201?00020110000?00?0????13010
01300?????0001000100001100 11[01]0002110000[01]?01211201?0??010000??000100200001 1
10121??221111000000000 0???2?????00000003310?00??0???0?????????????0?20?????????????

??
??
??
??0?????????????????????00
Vincelestes
21?2?111000[12]2021??1111010121130120011000??00?0000101?1000000000?00?0????130
1001300????0000002000000100110000210??000?0120?101?0??010000??0001012000011100
01??221100?00000000?1003000000100033334200003O??00????????????0?200111101001 1?11
0?1201?101?02110111100111010100011 10?00001?210122000000011100002110000?1020?00
?1001030201011000111010 0??0???210???0?00111002020011?00123?112400?1110100101110
10010011[01]2111111002011000000001100211 1??????21?0???????????010110100010201000
0001012000100020200010011100000 0??1???????????03000?0?2??000??0100???0???????????
?????00
Nanolestes
21?1011100012021??112?0??121030120011 1000?????????????00000?10000?00?0????130100
1300?????0001002100001100110000210 00?00?0121?1?1????0?0000??1012012000011 10121??
22111100000000010?1??????0010?02??0?00??0???0?????????????002??????????????????????
??
??
??
???00
Kielantherium
21?101110?012?21??111101?2????0130011?000?????????????00000?10000?00?0????1301101
300?????00011021100011011 1020151100 00[01]00120?102000?0100-0--
1002011101011?0121??2221110010000000???????????0???12??0??0??????0?????????????002?
??
??
??
???00
Aegialodon
???00???????????????????????????????????1?01??1300??
???00011021100011?1110201511 00?11001?????????????????????????????11???21??2221?100
10000000???002????????????????????????????????????
??
??
??
???0
Montanalestes
21?2?13100014021??111201032103013001 11000?????????????12020?10001000?0???13?2??
1300?????010210211000112012021151100111002????1?????????????????????????0???0?21??22
21?10011100000????????????????03??0?00????????????????????0?2?????????????????????????????
??
??
??1???
???0

Prokennalestes
21?1113100011021??1112010221030120011100??00?1[01]112?1?120200100000020?0????130
2201300?????020110111000112012021151100111002210212020?01001100101101000001120
121??2221210011100000?????????0101?0033?0000?00?1?0????????????0020???????????????
???
???23111251111110[12]00102?10?????13?1?11
1011????11001?????110?21????????2??1??????????????????????????????????1?????????????1??
??????????0??0
Murtoilestes
???0???????????????????00???????????1302201300??
????2021011100011?012021151100?1100?2102?2020?0?00110010110000000112?121??22212
1001?100000?????????0????????????????????????????????002??????????????????????????????
??
??
??
??0
Eomaia
21?1113100011021??11120102210301200111000??00?1011111?12010010000020?0????13022
01300?????020?10111000112??202?151100111??2210212020?0100110010??000?0001120121
??2221210011??000000010000000000003310010000?100????????????0020111?101001121100
1200?101102210111100211011120022102100010211122000000001?101102111000?012210002
12203020101301111101000001002100001200111?0?1????2??0????1??51?11??????????1?????
???????????????????????????11????????????????????????????0????????02??0??100?0?0?2???10?
?20???????02??1??????1?????????????00000?????????0?0?0??0?????10111000011?0
Juramaia 2?12-
?11000?????????1201022?0301100111000??00?1011201?1201001200?020?0????1302301300?
????02?????110?0112?1??2?1511001????2211212020?01001100101101110101120121??22212
1001?10000000010?0?001000003310000000?100-???-?-?-
???00201?11101001??1??0???????1102210??110?21?01????02210200???0???????????????????
???0?????????????????????????????????????
??0?
???000????????????0?0???????????????????????0
Kennalestes
21?2?1310001402110111200022???0?200111000??00?1111201?10020012000100?0????13022
01300?????020210211000112222031151100111002210212011?010011011010000?000112012
1??22212100111000001002????0010101[01]33200100001101????????????0020011??????????
???
2???????????????????????????????????110021221122102231112511??210211002[34]1?2001013?
????10110020100010010011002110??????21?2??????321300?0110200020200??00?010121211
000202201110021010?00??1011221??????????0??????????010?0????????????????????0
Asioryctes
21?2?131000140211011120002210301300111000??00?1112201?11020012000010?0????13022
01300?????020210211000112012031151100111002210212011?010011001000000?000112012
1??222121001110000000010000001010113321010000?101????????????00200111101??11?110
????????????????????????0?????0???0?11000??????2?0??00???????0??1?000?0122100021220
30201013011111010000000?020000?1?0011100212211221022311125?1??2102110?241?20010

13???1110110020100010010011002110??????21?2??????32130020110200020200?1001010?21
2110002022011100210100000??1011221??????30??0??????????0?000??0??????????????????0
Ukhaatherium
?1??????00?40??????????022???0??00?11000??00?1112201?1102001200002 0?0????1302201
300?????020?1021100011201203115110011 1?0221021201 1?01001100100000 0?0001120121??
22212100111000000001000000000111332101003 0?101????????????0020?11???1?011?1100??
0???0?10221011110?21101112002210 2100?1?210?2200000001?101102111000?012210002122
03020101301111101000 00???200???1??01??00?1?2??221???3??????????2??21????4?0????0???
????1????????0?0???1??????21?1??????????2????????21??0?0?????0????????00?010???21???0??
??????0??????0???????????????????0???????????????????????0?????????????????0
Zalambdalestes
21?2?1[13]1000[12]40211011120[01]02210301200111000??00?1112211?0212201200012 0?0??
??1302201300?????12031021100011022204205110001110220?102012?011011000000000?000
1120121??2221210011100000[23]00211?0001100[12][12]33[23]1010200?111????????????002
0???11010011?1100??0???01?0221011110?21101112002210210001021112200000001?101102
111210??1221000212203?201013?0113100000000002000001?001110031221122102231112 5?
1??210211002411200101 3???11101100201000100100110021 11??????21?2??????32130020110
200020201?100101012111100[01]20210110002101 0000??1011221?????0000000?2??000??010
00??0???????????????????
Daulestes
21?2?131000240211011120002210301200111000????????1??1??????0120???00?0????130220
1300?????020210211000 11?1120211511001110 0220?112021?0100111101 0000?0001120121?
?2221210011100000?00100??0001001133200?0??0?100????????????0020???????????????????
???
???????????????????????????110021201120102231112511???10?????2?????01013???????110?
201100??0?001????????1????2??1??????3???00?01?1??002?100?100?000?212110?[01]20???1?
?00??????00??2?????????????????????????????0?0??????????????????????0
Aspanlestes
?????1????0??0??????????1??????0???0?1?0?0??00?11112?1?1?010?100000?0?0????1302
311300?????110210211000 11?1120121511000 1100220?102032?1110312100?0000?10011201
21??2221210011100000??????????????0?033?0??0????1?0?????????????002?????????????
??
??3??????????1?0?0?1??
??0??????1?????????????????00?1???????????1????????????1?????????????????????????2?????
??
????????????0
Eoungulatum
?????1??????????????????1??????0???0?1???0??00?10110?1???????100??????0????1302421300
?????1103102110001 1?11201215111001200320?102232?11103121001 0000?100112000???222
1210011100000????????????????1332???0????1?0?????????????002??????????????????????????
??
??????????????????????????????????????3??????????1???0?1????0??????????????????????000????
?????????1????????????1??
??0
Cimolestes
2??2?1010?0240211?111200?22103012?0111000??00?10122?1?11010012000010?0????13023

11300????020210211000112212031151100112002210112111?010011110000000?000112012
1??2221210011100000???200??0?000?113321?00????1?0???????????20020?????????????????
???
???
0??
???0
Gypsonictops
2??2?10100024021 1?111200?22103012?0111000??00?11122?1?12120012000110?0????13023
11300????120210211000112212041151100112002210112111?010011110000000?000112012
1??2221210011100000????????0?000?113320?00????1?0????????????20020?????????????????
???
???
???
??0
Protungulatum
21?2?1010002402 11?1112000221030120011 1000??00?10120?1?12110010000010?0????13024
21300????1102102110001121120121511100120 1320?102232?11103100000000?100112000
1??2221210011100000????????0?0000113321?00????100???????????20020?????????????????
??222003?2122031 2?1
0130111312011????????????????????????????????2311125?1??1?0?0?00??[01]0?????1???????
???????11001??????1??21????????????2???1????
???0?????????????????0
Erinaceus
21?2?131000241211011120002210301300111000??00?1011011?12122002000100?0????13022
01300????11031021100011011202215111001211220?102122?111020??0000000?1003330001
??2221211111100000210300001001013333420002001110???????????20020111110100112110
01200?111102[12]10111101211011120022102100010211 1221?000001110111210121 0?122200
302122051201013011131201100000020010012001110031211120102231112511112102110023
113001113???1110110020110010010011002111213111212201110142130021111300020200 11
001101121211?022121011100211100201220112211 11??210001002?0000??01000??0?1110101
11000011?0
Leptictis
21?2?131000240211011120002210301300111000??00?1112211?12122002000100?0????13022
21300????11031021100011211204115110001101220?102111?110011110000000?100112000
1??2221211111100000210200000001011133210002 00?100????????????0020????????011?110
?1200?101??22??111?01211011120022 1021????02111221?000001110111221 1210?1222003?2
122?31201013011131201100???0200?001??01110031211120102231112511111102010023113
001113???11101100201100100100110021 10???????21?20?????32130121111200020300010011
01121211100202101110021010000122011221??????2?0000002?0000??0?0?0??0??????????????
???0
Canis
211??1310102402110111100022113013001110 00??00?0100011?0011010000001 0?1????11023
01200????00101000000000000000010210?001210000?001020?211011000110000?3101121001
??212100?021000000200200000000002332310000000100???????????200201111101 10112110
0120??101102120111101211111120022112100000211 1221?000001100110111 1110?22212020
21222051001013011 1??30110?0?002000001200111003021113110223111251 11?11?3??0?23111

101113?????101100200001000000110?112[01]2131112122011101421311201114010213000100110112110100220221210002111110012200122111 1??20000000200000110 10?[01]??0?111110110000011??
Felis
211??131010240211011110002211301300111010??00?0100011?0011010000001 0?1????1102301200?????0010100000000000000000210?001210000?001020?211000?00110000?3101121001??212100?02100???02002000000000013333430?00000100???????????20020111110110112110012 0??101102120111101211111200221121000002111221?00000110011011111 10?222120202122051001013011 1??30110?0?00200000120011100302111311022311125111?11?3??0?23111101113?????10110020000100000011 0?1120213111212201110142131120111401021300010011011211010022022121000211111001220012211 11??200000002000001 1010?0??0?111110110000011??
Rattus
2112?1110002412110111120002 22?0110002101 0???????????0??????0000??????0????13???21300?????13?000400000000????42041100013?1400??02022?211020???????????0033100?????2???????01?0????31?411112??2??333343???1?02112????????????200201111101001121100120 0?101102210111000110100021022112000010211121 1?001011110111211 1100?122100302122050001011011?31101101010001000012001110020101100201231112511111101000024110101113 1??2?1011001010011001002111111021311121220111014213002011121202140011001101121201102213212100021 21?20012111122111 1??200111000000000110 10?0??0?111110110000000??
Oryctolagus
2112?1110002402110111110002 22?011?1121010??00?0012000?001000000001???0????13???01300?????13?000400000000????210410?00?3?1300??02012?111030???????????01332000???2???????01?0????31?411112??2??333343??0??02112????????????211301111101001121100120 1?101102210111000200101120022102000 01?2111201?0010111101112101110?0?211130212205000101301013110110101000 1000?120011100201011 0020?23111251111110100002411010111 31??2?10110010000110010011101110213111212201110152130000111112120300 11?02111121201102213212100021 21?2001211111 22111 1??200111000000000111 10?0??0?111111110100?00??
Bradypus
211??131010241211011110002211301 3?0111000???????????0??????0000??????0??????????1500???????????????0??003???05540???????????????????????5??5????1??2???????00??????01??????????????301?011111111011211101 20??1011021201112102111111200221121 0000?2111321?1100011001101201100?12212010212205100101301 11??30110?0?0020000?120011100302111311022311125111?11?3??0?23021101013?????10110020000[01]000000110?112021311121220111014213113011110302101001?0110012110110220?2121000210??00112200122111 1??230???00200000110?100??0?1110111111113111??
Tamandua
211??1310102412110??????0?21?30???1??10?0??????????????????????????????0?????????????00???????????????0???0??5?05????1??2???????????????01??????????????3???0111111111011211101 20??1011021201112102111101200221121 0000?2111321?11000110011011011 00?22212010212205100101 30??1??30110?0?0020000?120011100312111311022311125111?11?3??0?231?1101013?????10110020010000000110?1120213111212201110142131130111103021010 02?0110212110110220?2121000210??00112200122111 1??230???00200000??0???0??0?1110111111113111??

Glyptotherium
211??13101024121101111000221130131011100??????????0??????0000??????0??????????1
500??????????????0??003???03330???????????
?????????5?05????10?2??????00??????01?????????????30100111111?1101121110120??101102
1201112002111101200221121000 0?2111321?110001100110120 1210?222120102122051001 01
30111??30110?0?0020000?12001111030211131102231112 5111?11?3??0?230?1101013?????1
0110020000?000000110?1120????11??2201110142131130111103021010010011001212111 02
20?2121010210 11?01122???22111???230??000200000??0?1?0??0?????1111011??????
Dasypus
211??13101024121101111000221130131011100??????????0??????0000??????0??????????1
500??????????????0??003???05540???????????
?????????5?05????10?2??????43??????01?????????????30100111111110112111 0120??101102
1201112002111101200221121000 0?2111321?110001100111110 1210?222120202122051001 01
30111??30110?0?0020000?12001111030211131102231112 5111?11?3??0?230[12]1101013????
?10110020000000000110?11202131112122011101421311301111130210100200110212121 11
0220?2121010210110011220012211 11??230??000?0000011010?0??0?1110111101131 11??
Chaetophractus
211??13101024121101111000221130131011100??????????0??????0000??????0??????????1
500??????????????0??003???05540???????????
?????????5?05????10?2??????43??????01?????????????30100111111110112111 0120??101102
1201112002111101200221121000 0?2111321?110001100111110 1210?222120202122051001 01
30111??30110?0?0020000?12001111130211131102231112 5111?11?3??0?23021101013?????1
0110020000[01]000000110?112[01]21311121220111014213113011111302101002001102121 2
1110220?212101021011001122001221111??230??000?00000??0?0?0??0?1110111101131 11??
Euphractus
21???13101024121101111000221130131011100??????????0??????0000??????0??????????15
00??????????????0??003???05540????????????
????????5?05????10?2??????43??????01?????????????30100111111110112111 0120??1011021
201112002111101200221121000 0?2111321?110001100111110 1210?222120202122051001013
0111??30110?0?0020000?12001111130211131102231112 5111?11?3??0?23021101013?????10
110020000[01]000000110?112[01]21311121220111014213113011111302101002001102121 21
110220?212101021011001122001221111??230??000?00000??0?0?0??0?1110111101131 11??
Holoclemensia
??????????????????????????????????0?1???0??????????????????110????????????1302101300?
????02011021100011111102115110 0?2100120?2?2011?11000100100111211001 12?121??2221
210010000000??????????????????????0??????????????????002???????????????????????????
???
???
???
???0
Sinodelphys
21???131010?4021??111?0??2210301300111000??00?00??111??1010110??00?0?0????1302??
1300?????0101100110001111 11?21151100121??1211212????110??????0110121100???0121??
2221?100100000000100100000000000113211120?10?100????????????0020???????001121?0012
0??1011?23101111012?111112?02211211110021????00000001?10??0?100000?01212010??12
042202011021?21100011011021000002001 1??0???

??0??????10??0???2????0??2?3?
?????0???1??????1??????????????00???????????0?0?0??0?????????????????0
Deltatheridium
21?2?14101022021??11110004210301300111000??00?0000111?00010110000000?0????13021
01300?????000110[02]11000111111021151100011001211202010?11000100100100102201120
121??2221210010000000100100??0000002222211100?12100???????????20020?????????????
??12??????????2????
???110212?1??00????????????????11100?020112????231112510??110100212312???1013????2?
10110?2?00001?????1101111???????2??1???????????02?11?2?002?2010100?000121???0?[01]
2031????002?011000??????????????1?0??0?????00???0?0????0?????????????????0
Atokatheridium
???????????2??????????????????????0????????00?????????????????????????????1?02101300??
???00011001100011?111021151100?0??0121?2?2010?1100010010010010220112?12???22212
10010000000??002??????????????????????????????
??
??
??
???00
Sulestes
21?2014101022021??111100142?1?01310111000??00?000011??00010110000000?0????13021
01300?????00021001100011111121151100122112211212010?1100010010011110220112012
1??22212100101000000????????????0?222221?10????1??????????????002????????????????????
??
??231112510??1?0????12?1?????????????????????
000?????????1?1??0??2???????????????
?0???0
Asiatherium
21?1?141010240211?111100142?0?01300111000??00?0000111?00010110000000?0????13022
01300?????010310211000110111122151101122112210212021?1111211?00010010100112012
1??22212100111000000??0?????000?0?222221010?????0??????????????0020??????1001121100?
?0????11?231011110?21101112002211211110021012200000001?101101100100?01?????????2
??????????????????????????????00?001110?3020112010?2311125????1111?0??23??4101013?
????10?10?2??0?01?001111???11???????2???????????????02011?2?00??21??100?0?0?2????0?[
01]20???11000??????10??2??????????????0????????????????????0??????????????????0
Kokopellia
?1?1?1410?022??1??1?1100?[34]2??????3?0111000??00?????1???0001011??00000?0????13021
01300?????01021021100011001112215110112210221?212020?1100111?10011110100112012
1??22212100111000000???1????0?000??222?1?10??0?100????????????2002???????????????????
???????????????????????????????????????0???
??????????????????????0??
??
???0
Anchistodelphys
????????????????????????????????????0????????00???????????????1?????????0????1?02101300??
???02031021100011?011122051101132102211212022?12102101010111102001120????22211
10011100000???002??????????????????????????????

??
??
??
??
Albertatherium
?????????????????????????????????0????????00???????????????1????????0?????302201300??
????????????0???1???1???????????????2112?2022?2110210110011111200??2?121??222111??
????0000?????????????????2????????????????????????002?????????????????????????????????
??
??
??
???
Didelphodon
21?2?14101021021??11110014211301?00111000??00?001???1?00011010000000?0????13022
01300?????000310211000112111122051201?321122112?2022?221020101002011021011 2012
1??2221110011100000??0??????0?000222221?00????1?0????????????20020??????????????????
??21201?1012031101
0110212211011??????????????????10?30201120102231112??0????1??0?1??11?????1???????1?
?????0?00?1???????0111?????????1?1????????????0?????????????????????????????????0???1????0
????1??1????????????????130000??????????0?0?0??0??????????????????
Pediomys
???2?14101021021??11110014211301?00111000??00???????1???????100????0?0????1302201
300?????010310211000111111122051201132112211212022?22102110000111101001120121?
?222111001110000???????????00?222221100??????0????????????20020????????????????????
??21201?101204220201
10212211011????????????????????????????????123111???0????0??0?1???1?????1??????1?????
???00?1???????0111????????????1??????????????????2???2?????????????????????????1?????????
???1???????????????????????????????????????0???????????????????
Turgidodon
???2?141010210211?111100?4?11301?00111000??00???????1?00010?10000000?0????130210
1300?????0103102110001111111321512011321122112120 22?2110211000011110200112012 1
??2221110011100000???????????000222221?00???21?0????????????2002???????????????????
??
?2??223111???0????0??0?1??11?????1??????1???????
?00?1???????0?11????????????1??1???????????
??0
Mayulestes
21?2?14101024?21101111001421030130011100 0??00?0000111?00010110000000?0????13021
01300?????0103102110001111111221512011311022112120 22?2200211000010110210112012
1??22211100111000000001000000000002222211001 11?100????????????002011111?10011?11
001201?1011?2310111101211111120022112 1?1???211122000000011101101110000?012?????
???204220201102121110 11??????210???0?0011100302011201112311125????110100??241101
01013?????101100210010100001111111111??????21?????????????020110200020211010 01000
?201010?020??01100021111100??2????????????130000002?0000?????????0?????????????????
0

Pucadelphys
21?2?14101024021101111001421030130011100 0??00?0000111?00010110000000?0????13021
01300?????120310211000110111122151201132102210212022?22112111000211111100112012
1??2221110011100000000100000000000222221101112100????????????00201111111001121 1
001201?1011?231011110121111112102211 2??????21112200000001110110111 0000?01213010
10120422020110212211011?101?0210???0?0011100302011201112311125 11??110100212411
0101013?????1011002100101000011111111??????21?1??????2????02011?200020211010011
1012010100020310110002101110 0?21011221?????1300000020000011 01000??0?????????????
????0
Andinodelphys
21?2?14101024021101111001421030130011100 0??00?0000111?00010110000000?0????13021
01300?????12031021100011211112215120103211221020202 2?22112111000211111100112012
1??22211100111000000001000000000002222211 10111?100????????????0020????????0???????
??0???0???0???????????????????2???????
?????????????????????????0???111003?201120112231112 5????1101002?2411010101 3?????10
110021001010000011111 11???????21?1????????????02?111?1002021?010011001201?10?0203
1011000210111 1??21????????????130000002?0000??0?0?0??0??????????????????0
Didelphis
21?2?14101024121101111001421030130011100 0??00?0000211?00010110000000?0????13021
01300?????02031021100011011112215120103211221020202 2?221120?00002111120011201 2
1??22211100111000000001000000000002222211 01112100???????????200201111111001 1?11
001201?10110231011110121111112[01]02211212110021112200000001110110111000000121
30101012042202111021231201111011021001002001110030201120111231112511111111 0021
2311[01]101013?????101100200000100011110111112131112121011100221200201112100202
10010011001201010002031001000211111101210112210 02??100000002000001101 00[01]??0?
101111132002000?0
Marmosa
21?2?14101024121101111001421030130011100 0??00?0000211?00010110000000?0????13021
01300?????020310211000111111121151201032122210202022?221120?000021111100112012
1??222111001110000000010000000100222221000111 2100????????????00201111111001121 1
001201?10110231011110121111112002211212110021112200000000111011011100000012130 1
0101204220211102122120111101102100100200111003020112011123111251111111100212 31
11101013?????10110020000010001111011111213111212101110022120020111210020210010
01101120101000203112110021111110121011221002??130000000000 00??0?000??0?1011????
?????????0
Caenolestes
21???14101024?21101111001421030130011100 0??00???????1??????0100????0?0?????302101
300?????020310211000111111132151201032122210202022?221120?0010210101001120121??
2221110011 1?00001001000000000012222210100202100????????0???002011111?100112110 01
201?101102310111101211111120022112121100211122000000011101101110000?01213020?0
120422010110212211011110000210100020011100302011 20?11231112511111113002?231101
01013?????10?10020000000002111011111 2131??211101110032120020111310020 21?010011?
01201010002032??10002111?220121???221002??1300000000000001 10?0?0??0?1011?????????
???0
Dasyurus
21?2?14101024021101111001421030130011100 0??00?00[01]0211?00010110000000?0????13

02101300?????0203102110001111111221512010321222112020222?221120?001021010200112
0121??22211100111000001002000000000133223110001121000????????????00201111111110011
211001201?10110231011110121111112002211212110021112200000001110110111000?0121
312010120510010121213?1101111000021010002001110030201120113231112511111130021
2310110101013?????10110020000010002111011121?131??2111011100421200201111120202111
0100110012010100020320110002111111012101122100210??1300000020000110100000??0?1011
?1132002000?0
Perameles
21???141010241211011110014210301300111000??00??????????????0000????0?0?????302101
300?????020210211000112111132151200032122210202022?221120?00102101020011201211??
222111001111?000000020000000000222221010021210 1????????????0020111111110011211001
201?10110231011110121111112002211212110021112200000001110110110010 0?01213020?1
120510020121213?110111101002001000201111003020112021123111251111111113002?231141
01013?????1011002000001000211101111213 1??211101110042120020111210020210010011
021201010002032121000211101101211???221001??230001000?0000??0?000??0?10111113200
2000?0
Dromiciops
2112?1410102402110111100142103013001110000??00?000021??00010000000000?0????13021
01300?????020310211000111011132051201032122211202022?221120?00000001110011201 2
1??222111001110000000001000000010122222101002121000????????????002011111?10011211
001201?101102310111101211111120022112121100211122000000011101101110000?0121302
0?1120510020121213?120111101002100100200111003020112011123111251111111113002?231
?4101013?????10110020000010002111001110213 1??21220111003213002011111002031101 0
01100120?0100020321211002101111012101122100 2??130000002000001 10?000??0?1011????
?????????
Thylacomyidae
211??14101024?211011110014210301300111000??00???????1??????1000????0?0?????30????
?00??????????????00011??11132??1????3???4?????2042???1120?00100001??0111201211??222
101???110000?0002000000???1222221??0?2?210?????????????00201111111?10011211001201?
10110231011110121111?11120022112111100211122000000011101101110000?012130?0???205
10020121213?1??11??0?002?0?000201111003 02?112?113231112511111113002?2310?101013
?????1011002?000010002?1???11??2131??21??0?1100421?0020111?1002021?010011?212?10
1?0020?2??1?0021?1???0121???22100???230001000?0000??0?000??0?1011?????????????
Macropus
211??141010240211011110014210301300111000??00??????????????0000????0?0?????3?2531
300?????1303002000001133011320413100431240 0?302042?211120?00100000?30011201211??
222101110110????200400001?????332232?110202112????1?1?0???002011111111001121100 12
01?101102310111101211111120022112111100211122001000011101101210010?01213120?11
2051001012121 3?11011110100200000020111100302111313032311125111111113002?2311410
1013?????101100200000100031110011112131??21220111005213002011111102131101001 10
21201010003032121100 2101?0[012]0121???221002??130001002000001111?000??0?10111113
2002000??
Acrobates
211??141010241211011110014210301300111000??00??????????????0000????0?0?????3?3531
300?????130210200000110101132151200042124 20?302042?211120?0010000111001120121??
222101001110????0003000000010?2222310000202110????????????002011111?1001121100 12

01?101102310111101211111120022112111110?21111220000000111011011110000?01213020?11
2051002012121213?12?111101002?0?1?0201111003021113130323111251111111113002?2311410
1013?????101100200000100011110111202131??212?11110052131020111310020311010010110
012010100020321211002111120121???221002??1300000000000000110?000??0?1011?????????
???0
Phascolarctos
211??131010240211011110014210301300110000??00??????????????0000????0?0?????3?3531
300?????1303202000001133011320413000431242 0?302042?211120?00100100?30111120121??
22210111011 0????2004000010????332233?20010211 1????????????00201111111001121 10012
01?101102310111101211111120022112111110?21111220010000111011011110000?01213020?11
2051002012121213?1101111010021001?02011210030211100103231112511111111113002?2311410
1013?????10110020000010002111011111 2131??2112011100321300201113110214100100111
01201010003032011000211110 20121???221002??1301110020000001101000??0?1011????????
?????
Vombatus
211??141010240211011110014210301300110000??00??????????????0000????0?0?????3?3531
300?????1303202000001133011320413100431242 0?302032?211120?00100000?30111120121??
22210111011 0????3004010120????332233?201?02112?????????????00201111111001121 10012
01?1011023101111012111111200221121111100211122001000011101101100100?01213020?11
2051002012121213?11011110100210010020112100302 1110?0032311125111111113002?2302310
1013?????10110020000010001111001111 2131??211201110052130020111310021411 0100110
0120101000303202100021111001121???221002??1301110020000001101000??0?1011????????
?????
Phalanger
211??141010240211011110014210301300111000??00??????????????0000????0?0?????3?3531
300?????130320200000113300132151200043124 20?302042?211120?00100100?30011120121??
22210111011 0????2003000000010?222222021020211 1????????????002011111110011211001
201?1011023101111012111111200221121111100211122000000001110110111 0000?01213020?1
12051002012121213?1201111010021001002011210030211131303231112511111111113002?231141
01013?????10110020000010003111001111 2131??21222111100521310201113110213100100 11
001201010003032021000211111 20121???221002??1301110020000001 10?000??0?1011???????
?????0
Pseudocheirus
211??141010240211011110014210301300111000??00??????????????0000??????0?????3?3531
300?????130320200000113300132041300042124 20?302042?211120?00100100?30111120121??
22210111011 0????2003000010010?33223200002021 11????????????002011111110011211001
201?101102310111101211111120022112111110121112200000000111011011110000?01213020?1
12051002012121213?1201111010021001002011210030211131303231112511111111113002?231141
01013?????1011002000001000311100112121 31??21222111100521310201112100203110100 11
0012010100120321210002111112 0121???221002??1300010000000001 10?000??0?1011???????
??????
Petauroides
211??141010240211011110014210301300111000??00??????????????0?00??????0?????3?3531
300?????130320200000113300132041300042124 20?302042?211120?00100100?30011120121??
22210111011 0????2003000000010?33223200002021 11????????????002011111110011211001
201?1011023101111012111111200221121111100211122000000001110110111 0000?01213120?1

1205100201212l3?120111101002100100201121003021113130323111251111111300 2?231141
01013????10110020000010003111001121213 1??2122111100521310201112100202110100 11
00120101001203212110021011120121???221002??1000000000000 00110?000??0?1011???????
??????

Supplementary Results 2. List of common synapomorphies of the node 172 (Ornithorhynchidae):

4(2), degree of development of Meckel’s sulcus, vestigial or absent.

31(1), gracile and elongate dentary peduncle, present.

34(1), gracile base of the coronoid process, present.

35(1), height of the coronoid process of the dentary, reduced.

170(1), development of the distal metacristid, absent.

557(1), second lower molar, neomorphic cusp (NC1) anterior to the hypoconulid, present.

558(1), lower molars with mid-valley present between the trigonid and taloned, mid-valley complete.

Supplementary Results 3. Measurements of *Patagorhynchus pascuali* (Holotype, MPM-PV-23087, right m2).

5.8 mm anteroposterior length

3.7 mm anterior transverse width

4.2 mm posterior transverse width

2.4 mm preserved crown height

1.6 mm anterior triakididrepanon anteroposterior length

1.8 mm posterior triakididrepanon anteroposterior length

3.2 mm posterior root height

3.3 mm. anterior root height

Supplementary references

1. Zhou, C. F., Bhullar, B. A. S., Neander, A. I., Martin, T., & Luo, Z. X. New Jurassic mammaliaform sheds light on early evolution of mammal-like hyoid bones. *Science* **365**, 276-279 (2019).

2. Pascual, R. et al. First discovery of monotremes in South America. *Nature* **356**, 704-706 (1992a). doi: 10.1038/356704a0

3. Pascual, R. et al. The first non-Australian monotreme: an early paleocene South American platypus (Monotremata, Ornithorhynchidae) in *Platypus and Echidnas* (ed. Augee, M.L.) 1-14 (Royal Zoological Society of New South Wales, 1992b).

4. Pascual, R., Goin, F. J., Balarino, M. L. & Udrizar Sauthier, D. E. New specimens of *Monotrematum sudamericanum*, and the convergent evolution of the triangulate molar. *Acta Palaeontol Polonica* **47**, 487-492 (2002).

5. Wood, C. B. & Rougier, G. W. Updating and recoding enamel microstructure in Mesozoic mammals: in search of discrete characters for phylogenetic reconstruction. *J Mammal Evol* **12**, 433-460 (2005). doi: 10.1007/s10914-005-6971-0

6. Woodburne, M. O. Monotremes as pretribosphenic mammals. *J Mamm Evol* **10**, 195-248 (2003). doi: 10.1023/B:JOMM.0000015104.29857.f0

7. Rowe, T., Rich, T. H., Vickers-Rich, P., Springer, M. & Woodburne, M. O. The oldest platypus and its bearing on divergence timing of the platypus and echidna clades. Proc Natl Acad Sci 105, 1238-1242 (2008). doi: 10.1073/pnas.0706385105

8. Rougier, G. W., Apesteguía, S. & Gaetano, L. C. Highly specialized mammalian skulls from the Late Cretaceous of South America. *Nature* **479**, 98-102 (2011). doi: 10.1038/nature10591

9. Krause, D. W. et al. Skeleton of a Cretaceous mammal from Madagascar reflects long-term insularity. *Nature* **581**, 421-427 (2020). doi: 10.1038/s41586-020-2234-8

10. Rich, T. H. et al. The mandible and dentition of the Early Cretaceous monotreme *Teinolophos trusleri*. *Alcheringa* **40**, 475-501 (2016). doi: 10.1080/03115518.2016.1180034

11. Goloboff, P. A., Farris, J. S., Nixon, K. C. TNT, a free program for phylogenetic analysis. *Cladistics* **24**, 774–786 (2008).

12. Goloboff, P. A. & Catalano, S. A. TNT version 1.5, including a full implementation of phylogenetic morphometrics. *Cladistics* **32**, 221–238 (2016). doi: 10.1111/cla.12160

13. Swofford, D. L. & Begle, D. P. PAUP User's Manual. Center for Biodiversity, Illinois Natural History Survey, Champaign, Illinois (1993).

14. Coddington, J. & Scharff, N. Problems with zero-length branches. *Cladistics* **10**, 415–423 (1994).

15. Bremer, K. The limits of amino acid sequence data in angiosperm phylogenetic reconstruction. *Evolution* **42**, 795–803 (1988).

16. Bremer, K.R. Branch support and tree stability. *Cladistics* **10**, 295–304 (1994).

17. Felsenstein, J. Confidence limits on phylogenies: an approach using the bootstrap. *Evolution* **39**, 783–791 (1985).

18. Goloboff, P., Farris, J. & Nixon, K. *T.N.T: Tree Analysis Using New Technology*. program available from the authors and www.zmuc.dk/public/phylogeny/tnt (2003).

19. Luo, Z-X., Kielan-Jaworowska, Z. & Cifelli, R. L. In quest for a phylogeny of Mesozoic mammals. *Acta Palaeontol Polonica* 47: 1-78 (2002).

20. Luo, Z-X., & Wible, J. R. A Late Jurassic digging mammal and early mammalian diversification. *Science* **308**, 103-107 (2005). doi: 10.1126/science.1108875

21. Luo, Z-X., Ji, Q. & Yuan, C. X. Convergent dental adaptations in pseudo-tribosphenic and tribosphenic mammals. *Nature* **450**, 93-97 (2007). doi: 10.1038/nature06221

22. Luo, Z-X., Yuan, C. X., Meng, Q. J. & Ji, Q. A Jurassic eutherian mammal and divergence of marsupials and placentals. *Nature* **476**, 442-445 (2011). doi: 10.1038/nature10291

23. Bi, S., Wang, Y., Guan, J., Sheng, X. & Meng, J. Three new Jurassic euharamiyidan species reinforce early divergence of mammals. *Nature* **514**, 579-584 (2014). doi: 10.1038/nature13718

24. Huttenlocker, A. K., Grossnickle, D. M., Kirkland, J. I., Schultz, J. A. & Luo, Z.-X. Late-surviving stem mammal links the lowermost Cretaceous of North America and Gondwana. *Nature* **558**, 108-112 (2018). doi: 10.1038/s41586-018-0126-y

25. Kielan-Jaworowska, Z., Crompton, A. W. & Jenkins F. A. The origin of egg-laying mammals. *Nature* **326**, 871-873 (1987). doi: 10.1038/326871a0

26. Davis, B. M. Evolution of the tribosphenic molar pattern in early mammals, with comments on the “dual-origin” hypothesis. *J Mammal Evol* **18**, 227-244 (2011). doi: 10.1007/s10914-011-9168-8

27. Kielan-Jaworowska, Z. & Cifelli, R. L. Primitive boreosphenidan mammal [?Deltatheroida] from the Early Cretaceous of Oklahoma. *Acta Palaeontol Polonica* **46**, 377-391 (2001).

28. Lopatin, A. & Averianov, A. *Kielantherium*, a basal tribosphenic mammal from the Early Cretaceous of Mongolia, with new data on the aegialodontian dentition. *Acta Palaeontol Polonica* **52**, 441–446 (2007).

29. Woodburne, M. O. & Tedford, R. H. The first Tertiary monotreme from Australia. *Am Mus Nov* **2588**, 1–11 (1975).
